# Supplementary material for: SeATAC: a tool for exploring the chromatin landscape and the role of pioneer factors
Source: Genome Biol. 2023 May 22;24:125. doi: 10.1186/s13059-023-02954-5 (PMC10204251; doi:10.1186/s13059-023-02954-5)
Supplement: Supplementary file 1 — Additional file 1: Fig. S1. SeAAQTAC corrects batch effects in fragment size distributions in ATAC-seq data. Fig. S2. SeATAC detects differential V-plot with different shift size in synthetic data. Fig. S3. The performance of SeATAC on different ATAC-seq quality indices. Fig. S4. SeATAC accurately predicted differentially expressed genes from ATAC-seq signals surrounding the transcription start sites. Fig. S5. SeATAC predicts histone modifications. Fig. S6. SeATAC detects Etv2 binding sites with increased chromatin accessibility during Etv2 induced EB differentiation and MEF reprogramming. Fig. S7. SeATAC detects Ascl1 binding sites with increased chromatin accessibility during Ascl1 induced MEF reprograming. Fig. S8. SeATAC detects OSK binding sites with increased chromatin accessibility during OSK induced reprograming. Fig. S9. Induction of Ascl1 causes both chromatin relaxation and closure at Ascl1 binding sites. Fig. S10. Induction of OSKcauses both chromatin relaxation and closure at OSK binding sites. Fig. S11. SeATAC detects Etv2 binding sites with significantly altered chromatin accessibility during the Etv2 induced reprogramming. [file 13059_2023_2954_MOESM1_ESM.pdf]

Fig. S1

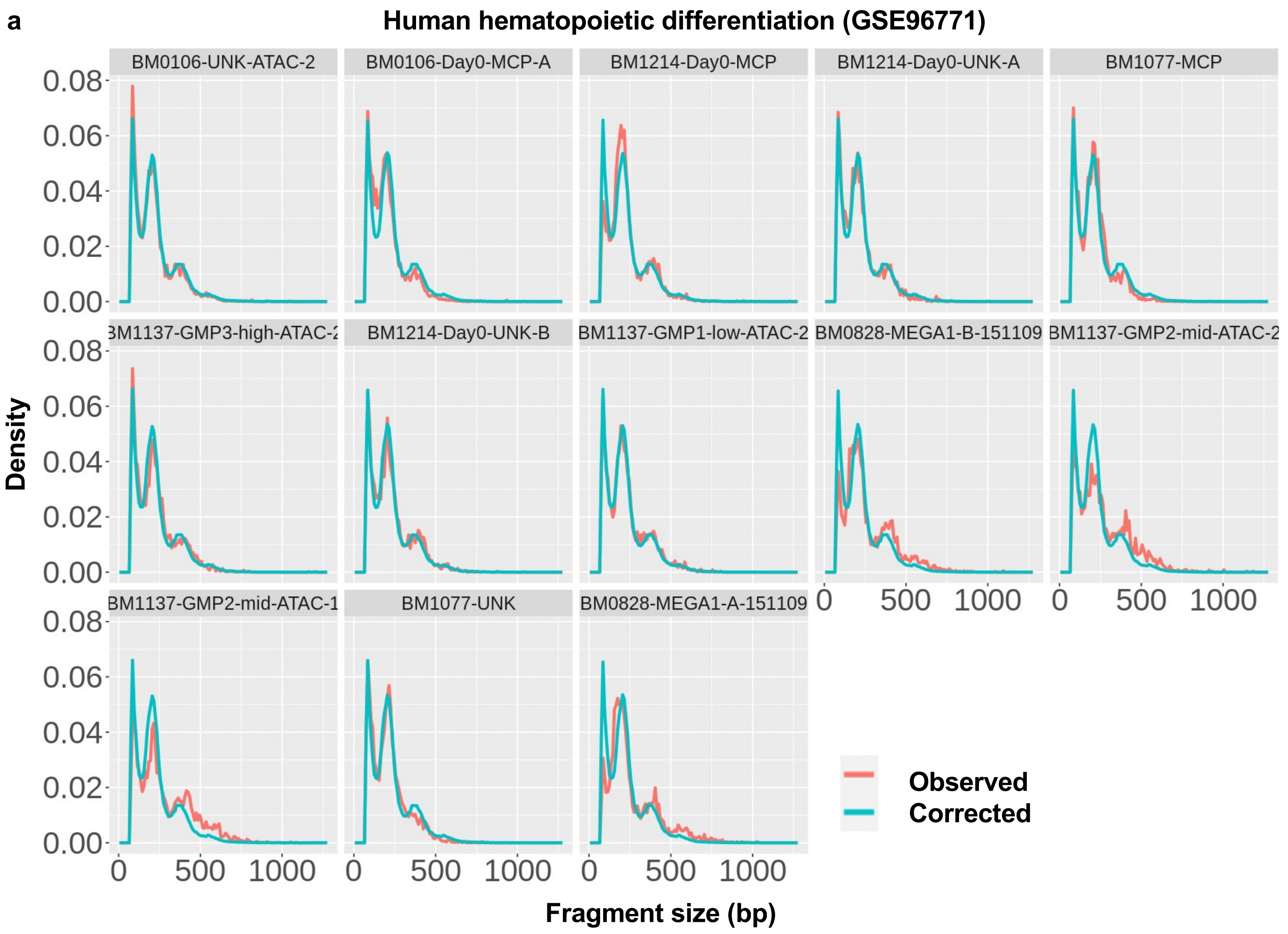
a
Human hematopoietic differentiation (GSE96771)
BM0106-UNK-ATAC-2
BM0106-Day0-MCP-A
BM1214-Day0-MCP
BM1214-Day0-UNK-A
BM1077-MCP
BM1137-GMP3-high-ATAC-2
BM1214-Day0-UNK-B
BM1137-GMP1-low-ATAC-2
BM0828-MEGA1-B-151109
BM1137-GMP2-mid-ATAC-2
BM1137-GMP2-mid-ATAC-1
BM1077-UNK
BM0828-MEGA1-A-151109
Density
0.08
0.06
0.04
0.02
0.00
0
500
1000
Fragment size (bp)
Observed
Corrected

Fig. S1. SeATAC corrects batch effects in fragment size distributions in ATAC-seq data. (a) The density plots show the observed (red) and corrected (green) fragment size distribution of 13 samples from a human hematopoietic differentiation ATAC-seq data (GSE96771). The x-axis shows the fragment size in base pairs (bp) and the y-axis shows the density.

Fig. S2

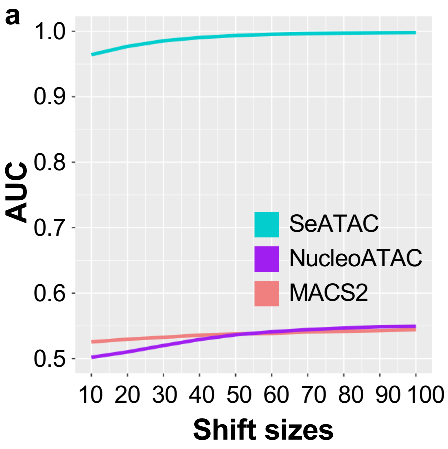
a
AUC
1.0
0.9
0.8
0.7
0.6
0.5
SeATAC
NucleoATAC
MACS2
10 20 30 40 50 60 70 80 90 100
Shift sizes

Fig. S2. SeATAC detects differential V-plot with different shift size in synthetic data. (a) The plot shows the AUC of SeATAC, NucleoATAC and MACS2 at different shift sizes (from 10 to 100) used to generate the synthetic data for evaluating task #1.

Fig. S3

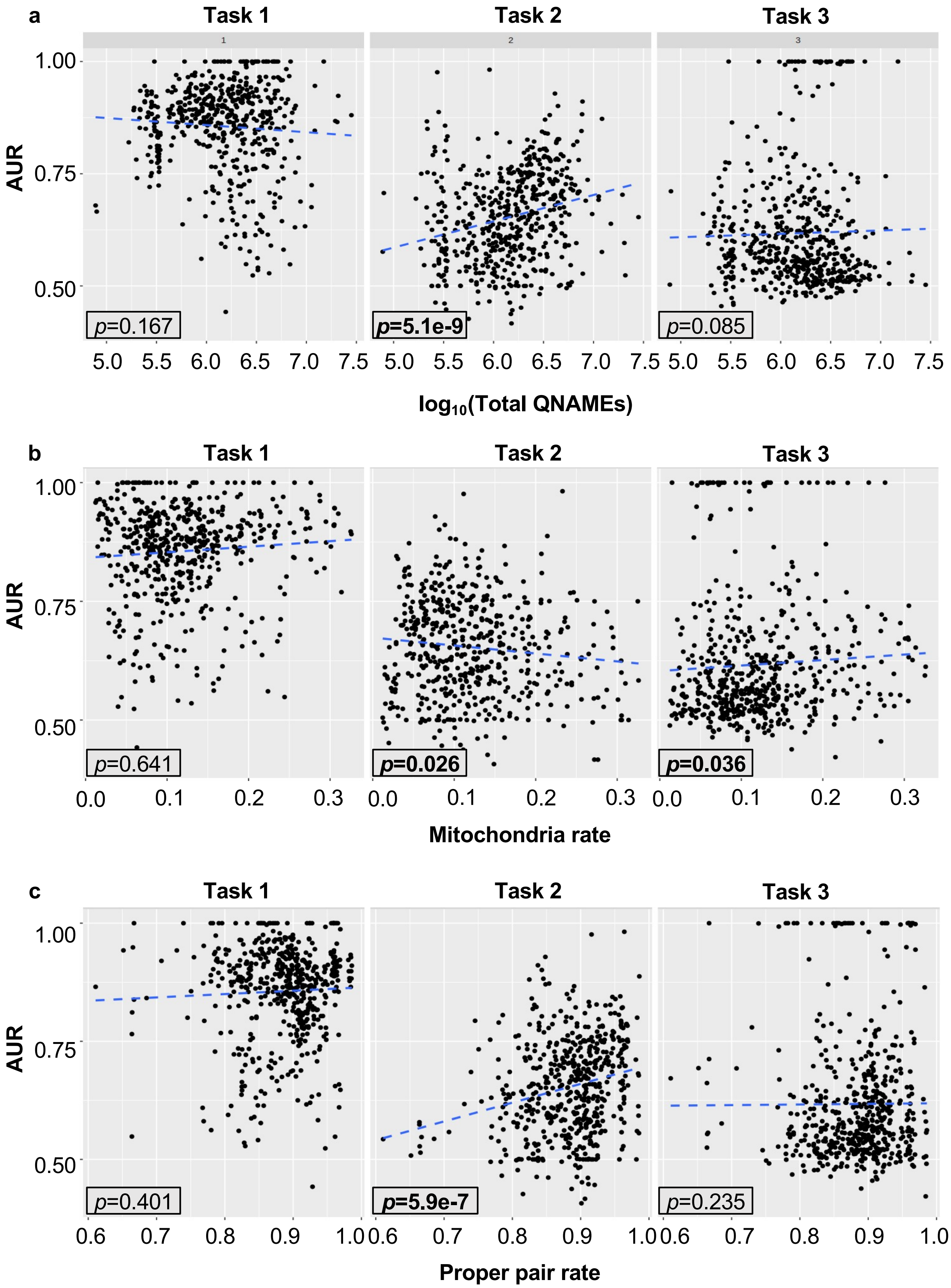
a
Task 1
Task 2
Task 3
AUR
1.00
0.75
0.50
p=0.167
p=5.1e-9
p=0.085
5.0 5.5 6.0 6.5 7.0 7.5
log10(Total QNAMEs)
b
p=0.641
p=0.026
p=0.036
0.0 0.1 0.2 0.3
Mitochondria rate
c
p=0.401
p=5.9e-7
p=0.235
0.6 0.7 0.8 0.9 1.0
Proper pair rate

Fig. S3

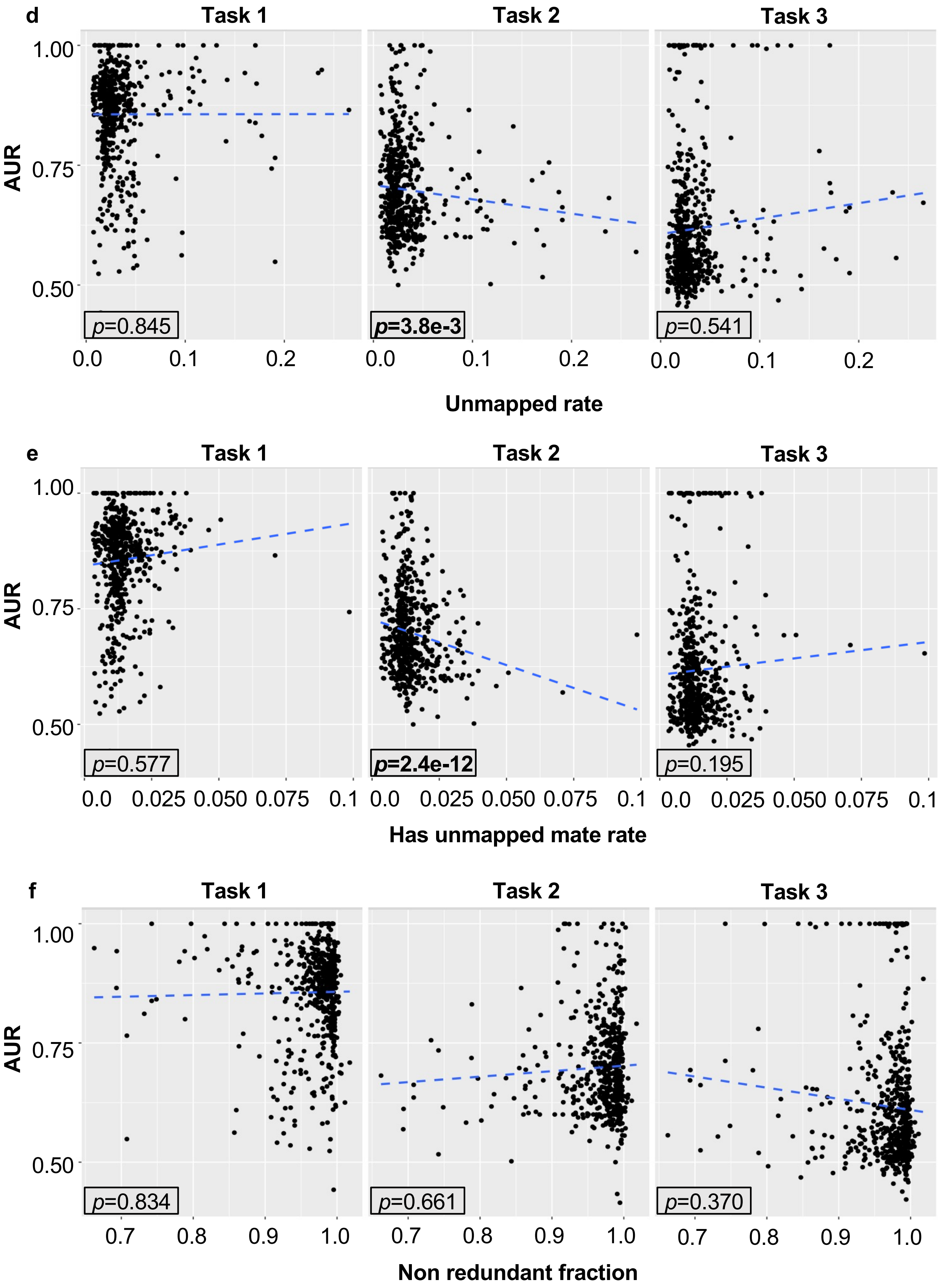
d
Task 1
Task 2
Task 3
AUR
1.00
0.75
0.50
p=0.845
p=3.8e-3
p=0.541
0.0
0.1
0.2
Unmapped rate
e
Task 1
Task 2
Task 3
AUR
1.00
0.75
0.50
p=0.577
p=2.4e-12
p=0.195
0.0
0.025
0.050
0.075
0.1
Has unmapped mate rate
f
Task 1
Task 2
Task 3
AUR
1.00
0.75
0.50
p=0.834
p=0.661
p=0.370
0.7
0.8
0.9
1.0
Non redundant fraction

Fig. S3

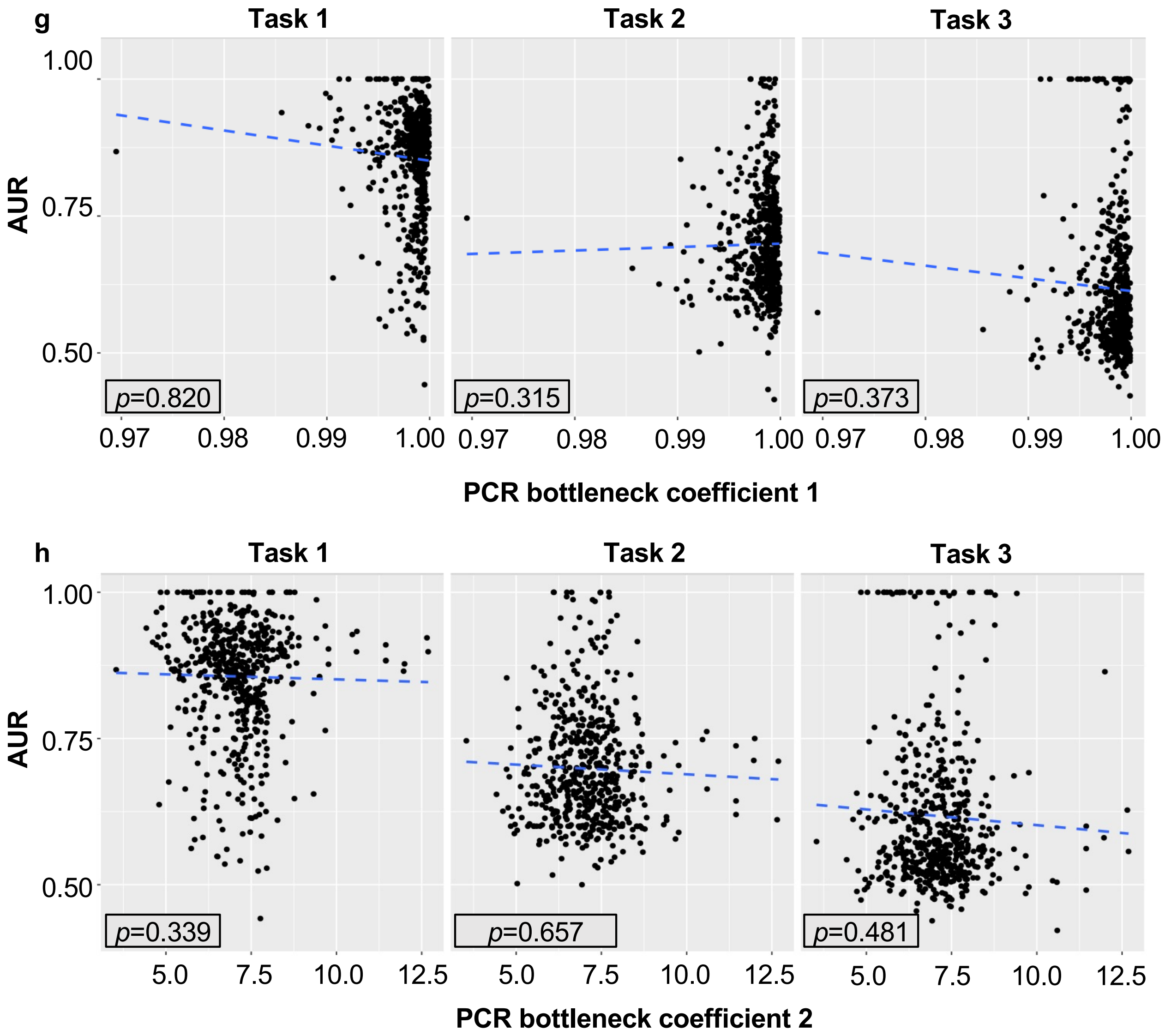

g
Task 1
Task 2
Task 3
1.00
0.75
0.50
AUR
p=0.820
p=0.315
p=0.373
0.97
0.98
0.99
1.00
PCR bottleneck coefficient 1
h
Task 1
Task 2
Task 3
1.00
0.75
0.50
AUR
p=0.339
p=0.657
p=0.481
5.0
7.5
10.0
12.5
PCR bottleneck coefficient 2

Fig. S3. The performance of SeATAC on different ATAC-seq quality indices. (a-h) The plots show the AUC (area under ROC) of SeATAC on 523 ATAC-seq samples from 20 studies at **(a)** total read counts (Total QNAMEs), **(b)** mitochondria rate, **(c)** proper pair rate, **(d)** unmapped rate**, (e)** has unmapped mate rate, **(f)** non-redundant fraction, **(g)** PCR bottleneck coefficient 1, and **(h)** PCR bottleneck coefficient 2.

Fig. S4

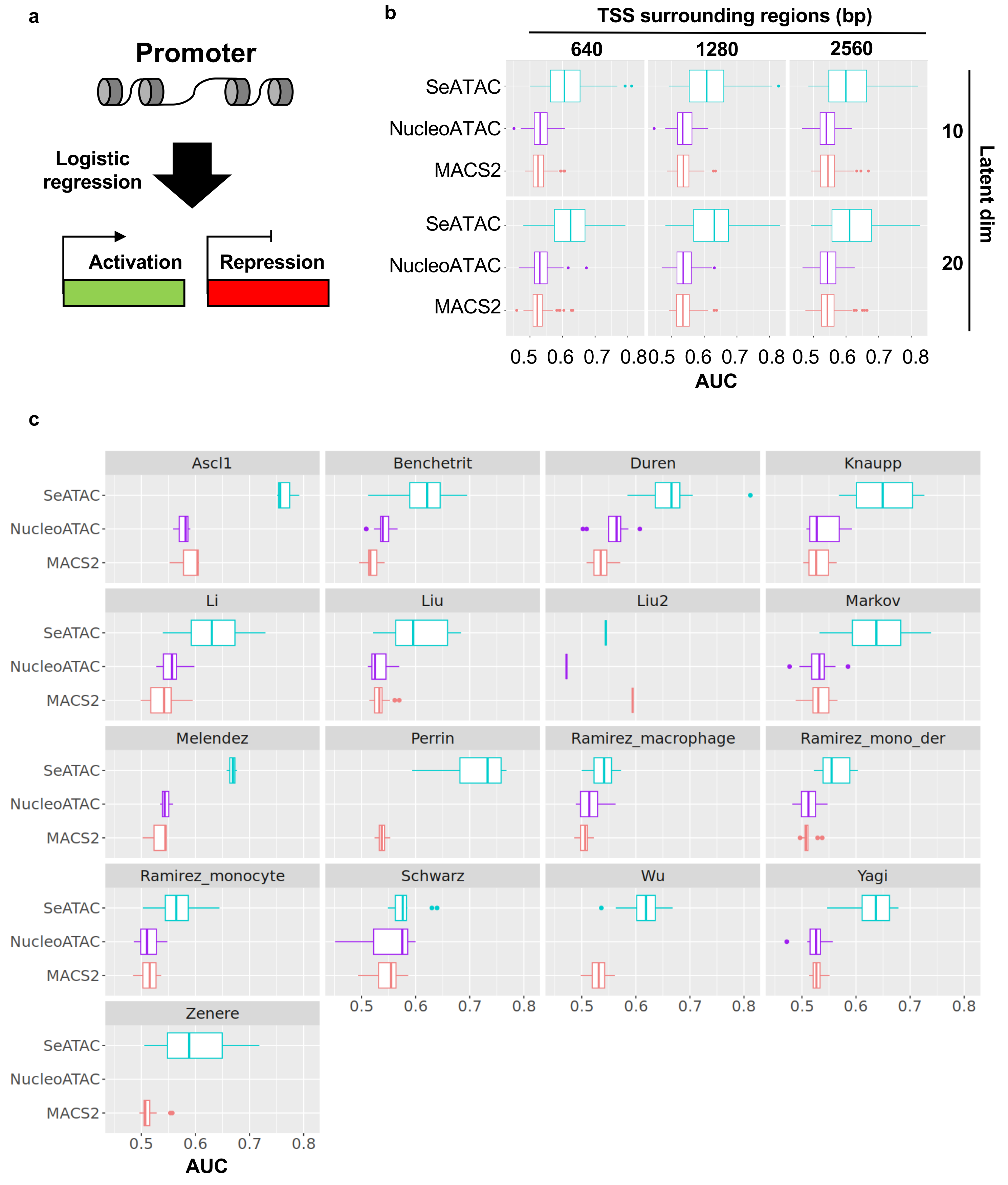
a
Promoter
Logistic regression
Activation
Repression
b
TSS surrounding regions (bp)
640
1280
2560
SeATAC
NucleoATAC
MACS2
10
20
Latent dim
0.5 0.6 0.7 0.8
AUC
c
Ascl1
Benchetrit
Duren
Knaupp
Li
Liu
Liu2
Markov
Melendez
Perrin
Ramirez_macrophage
Ramirez_mono_der
Ramirez_monocyte
Schwarz
Wu
Yagi
Zenere
SeATAC
NucleoATAC
MACS2
0.5 0.6 0.7 0.8
AUC

Fig. S4. SeATAC accurately predicted differentially expressed genes from ATAC-seq signals surrounding the transcription start sites. (a) The schematic shows predicting differentially expressed genes from the ATAC-seq signals over the promoter region. The differentially expressed genes were determined by the paired RNA-seq data. **(b)** The area under ROC (AUC) of three tools, SeATAC, NucleoATAC and MACS2 on the regions over promoter region (column wise) and latent dimensions (row wise). **(c)** The area under ROC (AUC) of three tools on 17 paired RNA-seq / ATAC-seq datasets.

Fig. S5

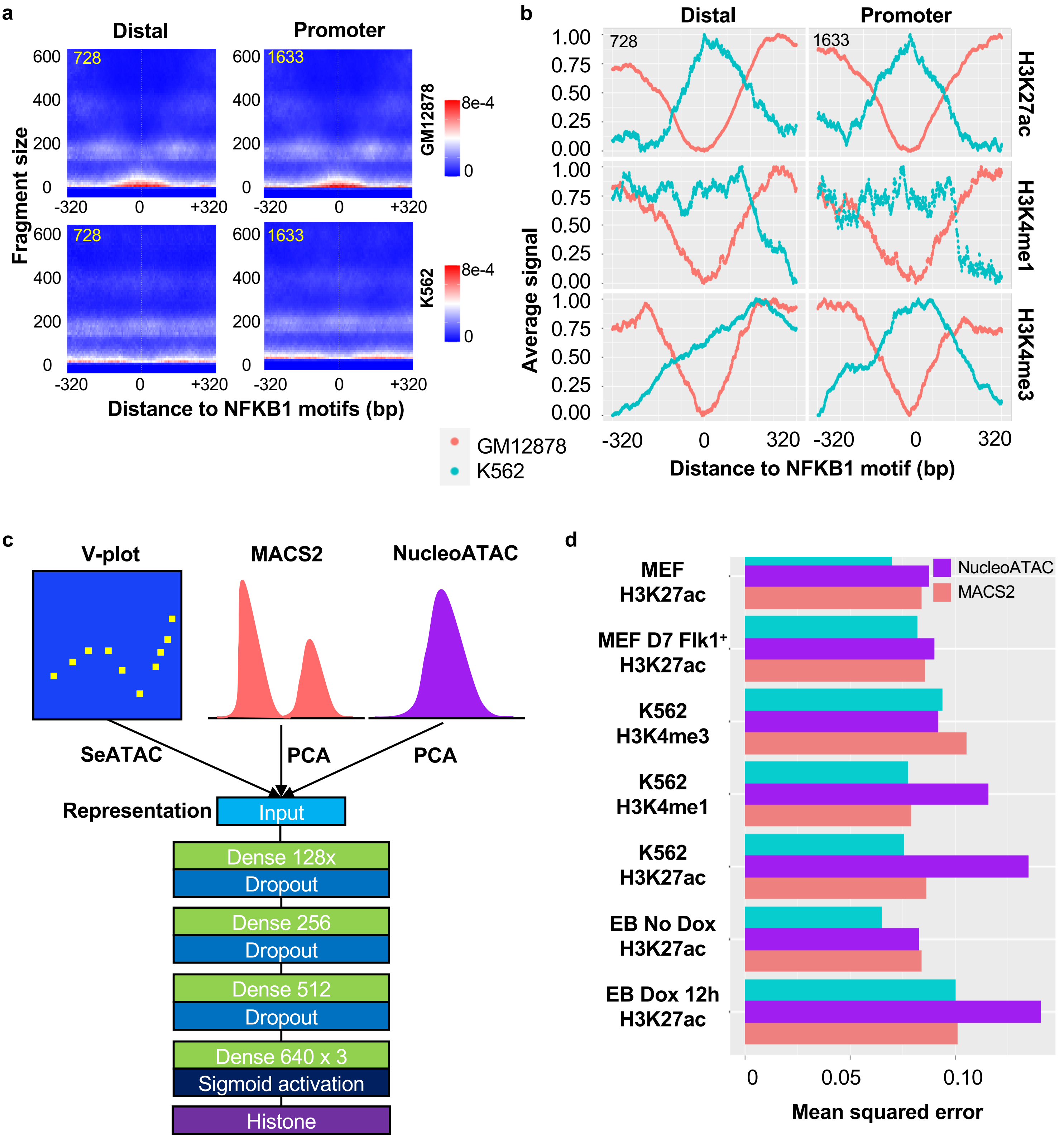

a
Distal
Promoter
Fragment size
600
400
200
0
728
1633
GM12878
K562
8e-4
0
-320
0
+320
Distance to NFKB1 motifs (bp)
b
Distal
Promoter
728
1633
1.00
0.75
0.50
0.25
0.00
Average signal
H3K27ac
H3K4me1
H3K4me3
GM12878
K562
-320
0
320
Distance to NFKB1 motif (bp)
c
V-plot
MACS2
NucleoATAC
SeATAC
PCA
PCA
Representation
Input
Dense 128x
Dropout
Dense 256
Dropout
Dense 512
Dropout
Dense 640 x 3
Sigmoid activation
Histone
d
MEF
H3K27ac
MEF D7 Flk1⁺
H3K27ac
K562
H3K4me3
K562
H3K4me1
K562
H3K27ac
EB No Dox
H3K27ac
EB Dox 12h
H3K27ac
NucleoATAC
MACS2
0
0.05
0.10
Mean squared error

Fig. S5. SeATAC predicts histone modifications. (a) The aggregated V-plot includes: 728 and 1,633 NFKB1 binding sites with increased chromatin accessibility in GM12878 compared with K562 at distal and promoter regions, respectively. The heatmap color indicates the estimated read density. **(b)** The line plots include: mean signal of H3K27ac, h3K4me1, H3K4me3 signals of 728 and 1,633 NFKB1 binding sites with increased chromatin accessibility in GM12878 compared with K562 at distal and promoter regions. **(c)** The schematic shows training a multilayer perceptron (MLP) to predict histone modifications from the ATAC-seq representations produced by V-plot, MACS2 and NucleoATAC. **(d)** The mean squared error of observed and predicted histone modification signals.

Fig. S6

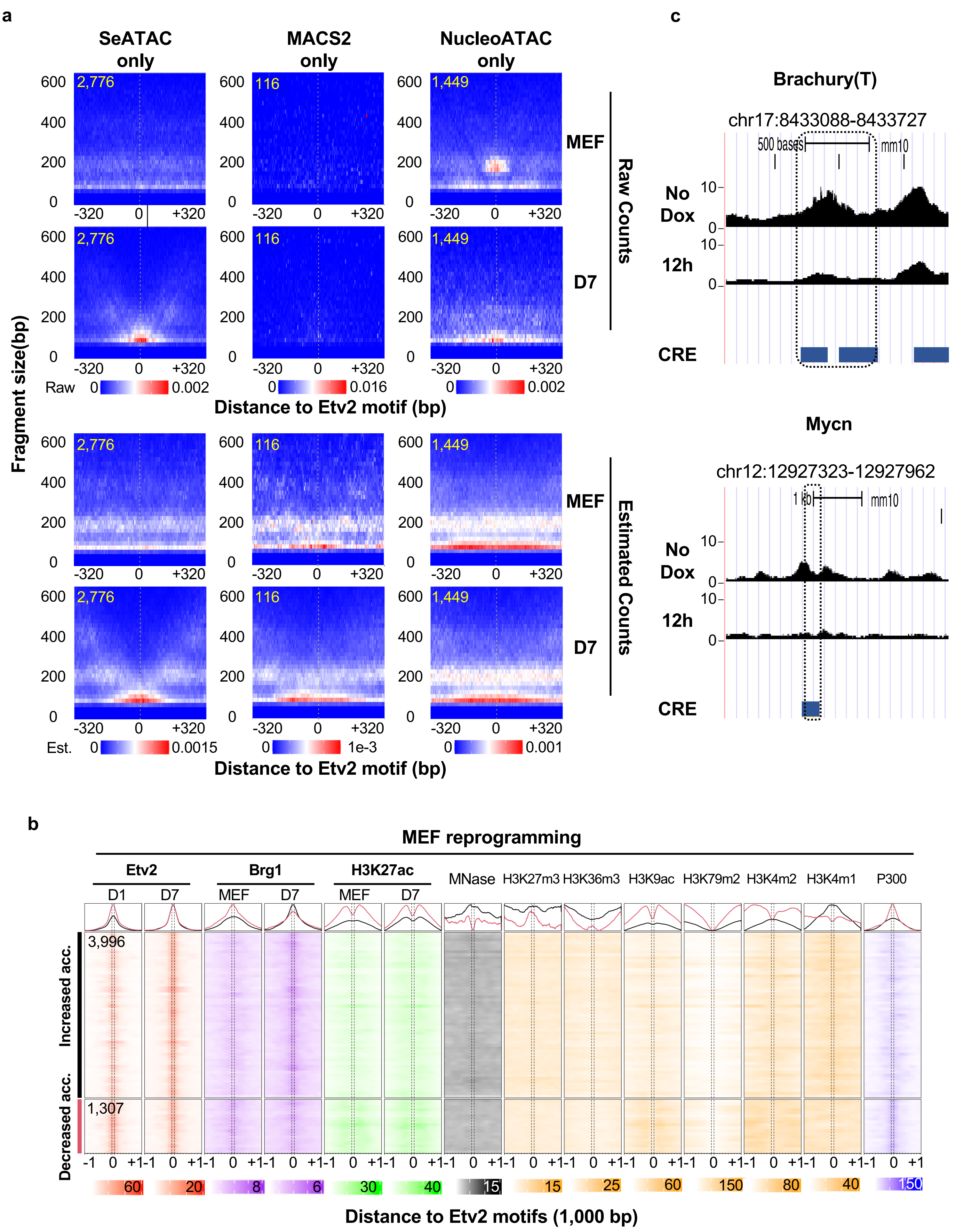

a
SeATAC only
MACS2 only
NucleoATAC only
2,776
116
1,449
MEF
D7
Raw Counts
Estimated Counts
Fragment size(bp)
600
400
200
0
-320
0
+320
Raw 0 0.002
0 0.016
0 0.002
Distance to Etv2 motif (bp)
Est. 0 0.0015
0 1e-3
0 0.001
Distance to Etv2 motif (bp)
b
MEF reprogramming
Etv2
Brg1
H3K27ac
D1
D7
MEF
D7
MEF
D7
MNase
H3K27m3
H3K36m3
H3K9ac
H3K79m2
H3K4m2
H3K4m1
P300
Increased acc.
Decreased acc.
3,996
1,307
-1 0 +1
60
20
8
6
30
40
15
15
25
60
150
80
40
150
Distance to Etv2 motifs (1,000 bp)
c
Brachury(T)
chr17:8433088-8433727
500 bases
mm10
No Dox
12h
CRE
10
0
Mycn
chr12:12927323-12927962
1 kb
mm10

Fig. S6. SeATAC detects Etv2 binding sites with increased chromatin accessibility during Etv2 induced EB differentiation and MEF reprogramming. (a) The aggregated V-plot includes 2,776, 116 and 1,449 Etv2 motifs with increased chromatin accessibility identified by SeATAC only, MACS2 only and NucleoATAC only using the ATAC-seq dataset of Etv2 induced MEF reprogramming (undifferentiated MEFs vs. Flk1^{+} cells at 7 days post-induction). Both raw V-plots and estimated V-plots are shown. The heatmap color indicates the normalized read density for raw counts (top) and the estimated read density for estimated read counts (bottom).
(b) The heatmaps show the Etv2, Brg1, H3K27ac ChIP-seq of 3,996 and 1,307 Etv2 binding sites that have increased (NOR->NFR) or decreased (NFR->NOR) chromatin accessibility in undifferentiated MEFs (Brg1 and H3K27ac), 1 day post-Etv2 induction (Etv2), and 7 days post-Etv2 induction (Etv2, Brg1 and H3K27ac). The heatmaps also include the MNase-seq, H3K27m3, H3K36m3, H3K9ac, H3K79me2, H3K4me2, H3K4me1 and P300 ChIP-seq from undifferentiated MEFs. **(c)** The UCSC genome browser track show the ATAC-seq density near the Etv2 motifs at the promoters of Brachyury (T) and Mycn. The Etv2 motifs become less accessible during the EB differentiation and both Brachyury (T) and Mycn are significantly down-regulated during Etv2 induced EB differentiation.

Fig. S7

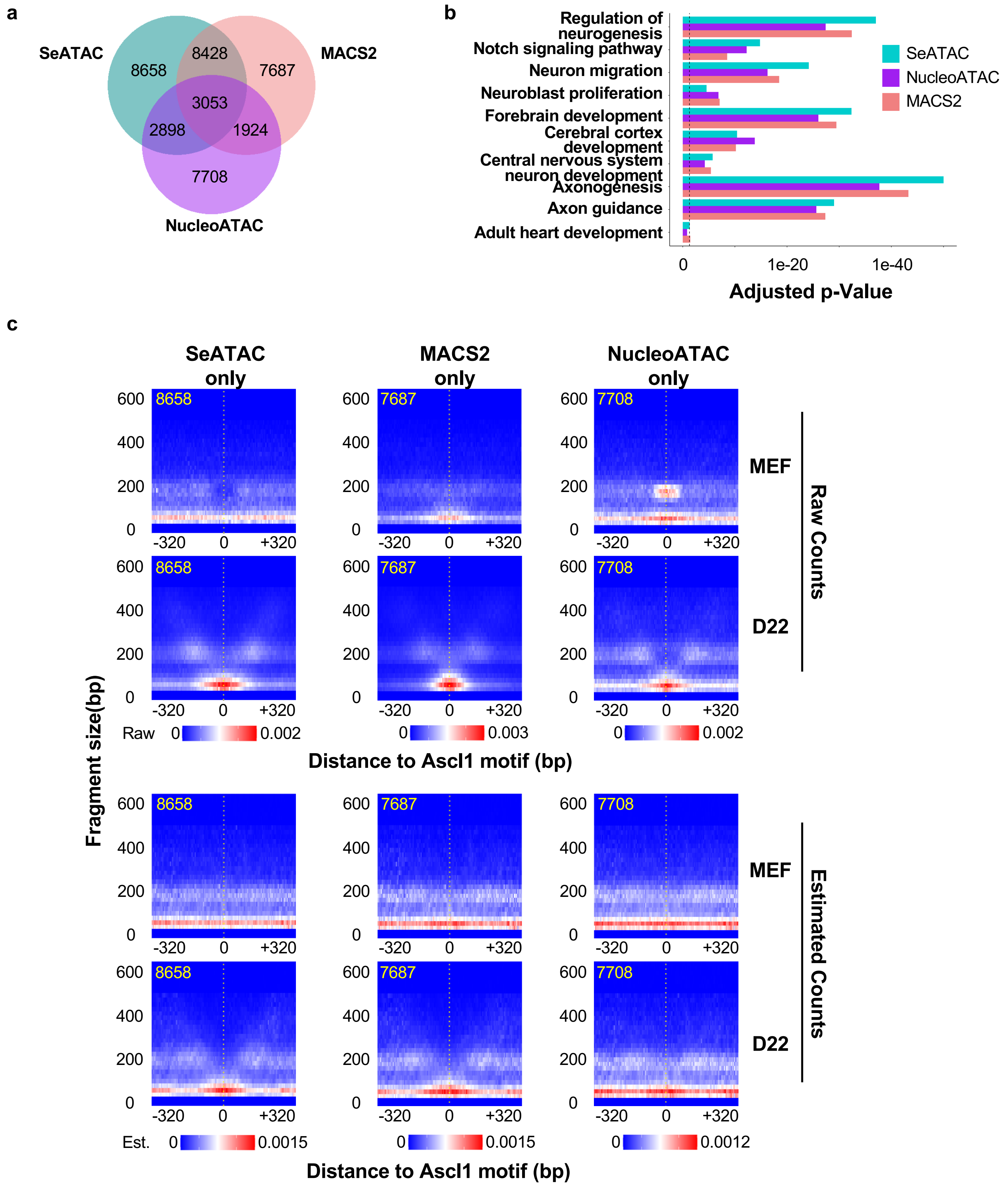

a
SeATAC
MACS2
NucleoATAC
8658
8428
7687
3053
2898
1924
7708
b
Regulation of neurogenesis
Notch signaling pathway
Neuron migration
Neuroblast proliferation
Forebrain development
Cerebral cortex development
Central nervous system neuron development
Axonogenesis
Axon guidance
Adult heart development
0
1e-20
1e-40
Adjusted p-Value
SeATAC
NucleoATAC
MACS2
c
SeATAC only
MACS2 only
NucleoATAC only
MEF
D22
Raw Counts
Estimated Counts
Fragment size(bp)
Distance to Ascl1 motif (bp)
Raw 0 0.002
0 0.003
0 0.002
Est. 0 0.0015
0 0.0015
0 0.0012
-320
0
+320

Fig. S7. SeATAC detects Ascl1 binding sites with increased chromatin accessibility during Ascl1 induced MEF reprograming. (a) The Venn diagrams show the number of Ascl1 motifs with increased chromatin accessibility identified using SeATAC, MACS2 and NucleoATAC. **(b)**The barplots show the Gene Ontology (GO) terms that are significantly associated with the genes that have promoters (-5,000 - +1,000bp region flanking the TSS) with Ascl1 binding motifs with increased chromatin accessibility, identified by SeATAC, MACS2 and NucleoATAC. The y-axis showed the adjusted *p*-value of the pathway analysis. **(c)** The aggregated V-plot includes 8,658, 7,687 and 7,708 Ascl1 motifs with increased chromatin accessibility identified using SeATAC only, MACS2 only and NucleoATAC only in ATAC-seq data of Ascl1 induced MEF reprogramming (undifferentiated MEFs vs. 22 days post Ascl1 induction). Both raw V-plots and estimated V-plots are shown. The heatmap color indicates the normalized read density for raw counts (top) and the estimated read density for estimated read counts (bottom).

Fig. S8

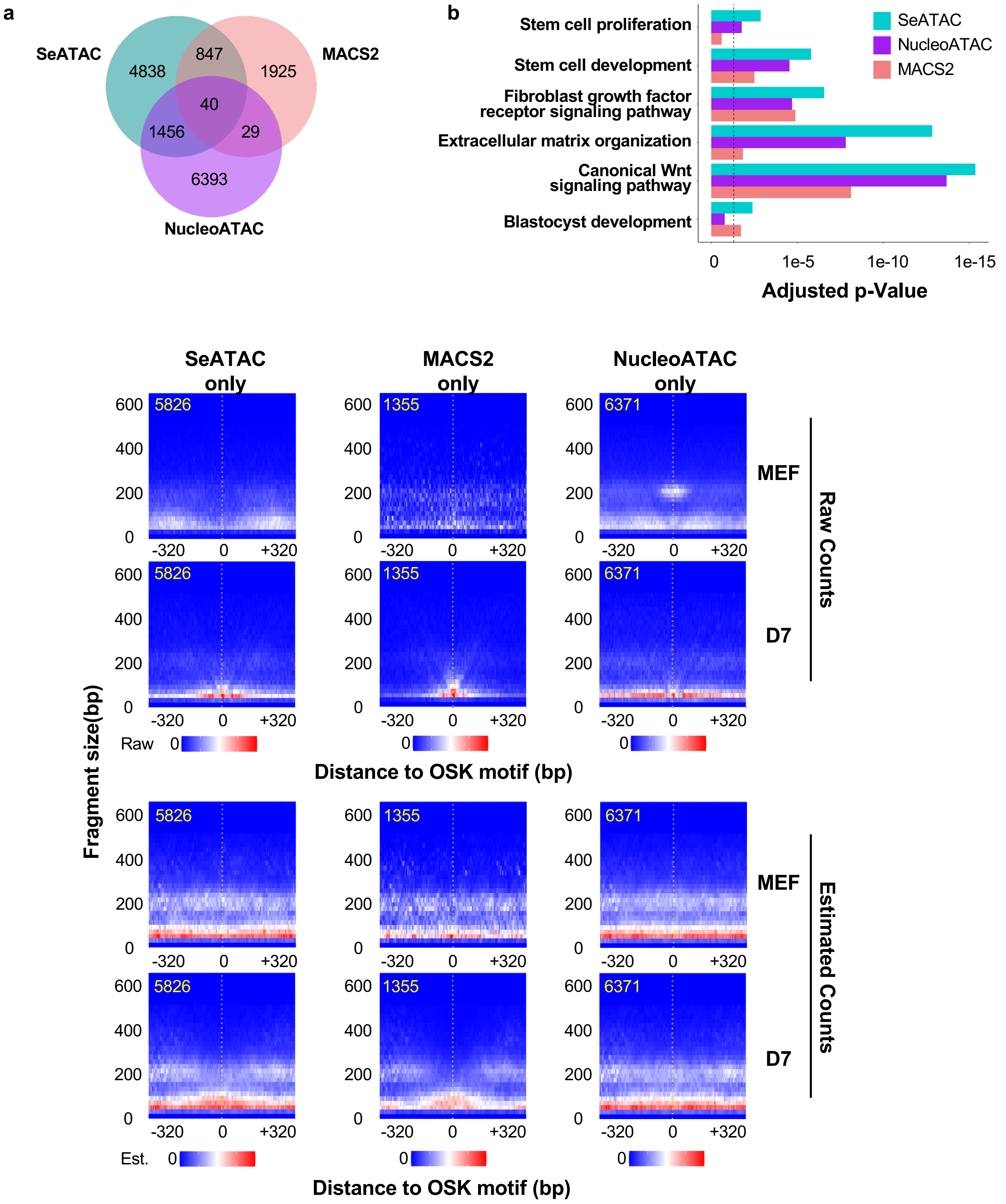
a
SeATAC
MACS2
NucleoATAC
4838
847
1925
40
1456
29
6393
b
Stem cell proliferation
Stem cell development
Fibroblast growth factor receptor signaling pathway
Extracellular matrix organization
Canonical Wnt signaling pathway
Blastocyst development
SeATAC
NucleoATAC
MACS2
0
1e-5
1e-10
1e-15
Adjusted p-Value
SeATAC only
MACS2 only
NucleoATAC only
5826
1355
6371
600
400
200
0
-320
0
+320
MEF
D7
Raw Counts
Raw
Fragment size(bp)
Distance to OSK motif (bp)
Estimated Counts
Est.
Distance to OSK motif (bp)

Fig. S8. SeATAC detects OSK binding sites with increased chromatin accessibility during OSK induced reprograming. (a) The Venn diagrams show the number of OSK motifs with increased chromatin accessibility identified using SeATAC, MACS2 and NucleoATAC. **(b)**The barplots show the Gene Ontology (GO) terms that are significantly associated with the genes with promoters (-5,000 - +1,000bp region flanking the TSS) that have OSK motifs with increased chromatin accessibility, identified by SeATAC, MACS2 and NucleoATAC. The y-axis showed the adjusted *p*-value of the pathway analysis. **(c)** The aggregated V-plot includes 5,826, 1,355 and 6,371 OSK motifs with increased chromatin accessibility identified using SeATAC only, MACS2 only and NucleoATAC only in ATAC-seq data of OSK induced MEF reprogramming (undifferentiated MEFs vs. 7 days post OSK induction). Both raw V-plots and estimated V-plots are shown. The heatmap color indicates the normalized read density for raw counts (top) and the estimated read density for estimated read counts (bottom).

Fig. S9

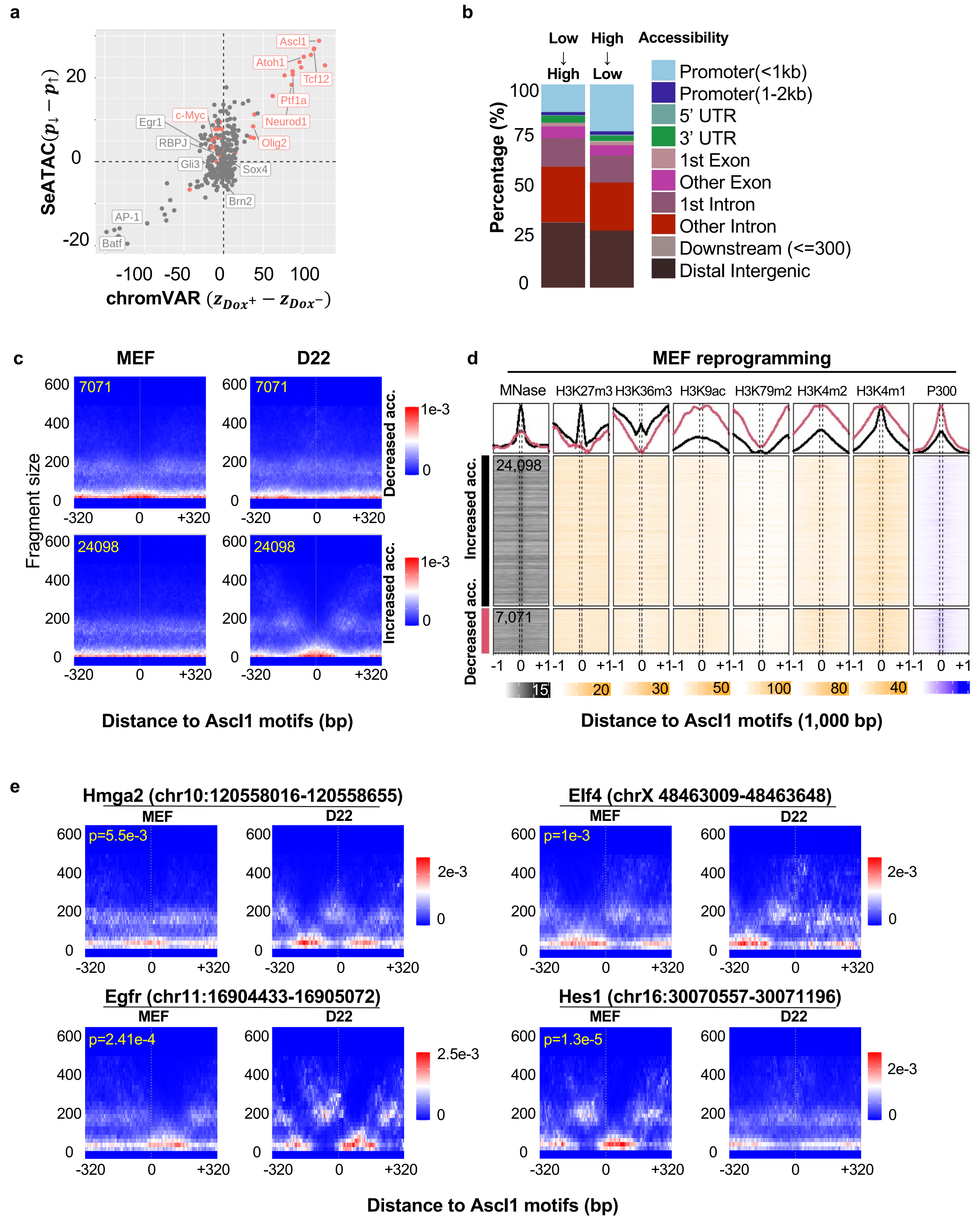

a
SeATAC($p_{\downarrow} - p_{\uparrow}$)
chromVAR ($z_{Dox^+} - z_{Dox^-}$)
Ascl1
Atoh1
Tcf12
Ptf1a
Neurod1
Olig2
c-Myc
Egr1
RBPJ
Gli3
Sox4
Brn2
AP-1
Batf
b
Low → High
High → Low
Accessibility
Percentage (%)
Promoter(<1kb)
Promoter(1-2kb)
5' UTR
3' UTR
1st Exon
Other Exon
1st Intron
Other Intron
Downstream (<=300)
Distal Intergenic
c
MEF
D22
Fragment size
7071
24098
Decreased acc.
Increased acc.
1e-3
Distance to Ascl1 motifs (bp)
d
MEF reprogramming
MNase
H3K27m3
H3K36m3
H3K9ac
H3K79m2
H3K4m2
H3K4m1
P300
24,098
7,071
Increased acc.
Decreased acc.
Distance to Ascl1 motifs (1,000 bp)
e
Hmga2 (chr10:120558016-120558655)
Elf4 (chrX 48463009-48463648)
Egfr (chr11:16904433-16905072)
Hes1 (chr16:30070557-30071196)
MEF
D22
p=5.5e-3
p=1e-3
p=2.41e-4
p=1.3e-5
2e-3
2.5e-3
Distance to Ascl1 motifs (bp)

Fig. S9. Induction of Ascl1 causes both chromatin relaxation and closure at Ascl1 binding sites. (a) The dot plots compare the changes of motif associated chromatin accessibility estimated by chromVAR (x-axis) and the difference of the percent of TFBS with decreased or increased chromatin accessibility estimated by SeATAC (y-axis). $\boldsymbol{z_{Dox^+}}$ and $\boldsymbol{z_{Dox^-}}$ are the normalized deviation score of Dox^+ condition (22 days post-induction for MEF reprogramming) and Dox^- condition (undifferentiated MEFs). $\boldsymbol{p}_{\downarrow}$ and $\boldsymbol{p}_{\uparrow}$ are the percent of TFBS that shows decreased or increased chromatin accessibility in Dox^+ condition compared with the Dox^- condition. **(b)** The barplots show the genomic distribution of Ascl1 binding sites with decreased (NFR->NOR) or increased (NOR->NFR) chromatin accessibility in MEF reprogramming. The change of chromatin accessibility is estimated using SeATAC. **(c)** The aggregated V-plot includes: 24,098 and 7,071 Ascl1 binding sites that have increased (NOR->NFR) or decreased (NFR->NOR) chromatin accessibility during MEF reprograming. The heatmap color indicates the estimated read density. **(d)** The heatmaps show the MNase-seq, H3K27m3, H3K36m3, H3K9ac, H3K79me2, H3K4me2, H3K4me1 and P300 ChIP-seq signals in undifferentiated MEFs of 24,098 and 7,071 Ascl1 binding sites that have increased (NOR->NFR) or decreased (NFR->NOR) chromatin accessibility during the MEF reprogramming. The change of chromatin accessibility is estimated using SeATAC. **(e)** The V-plot show Ascl1 motifs with decreased chromatin accessibility at the promoters (-5,000 - +1,000bp region flanking the TSS) of four genes (Hmga2, Elf4, Egfr and Hes1) that are down-regulated during the Ascl1 induced MEF reprogramming.

Fig. S10

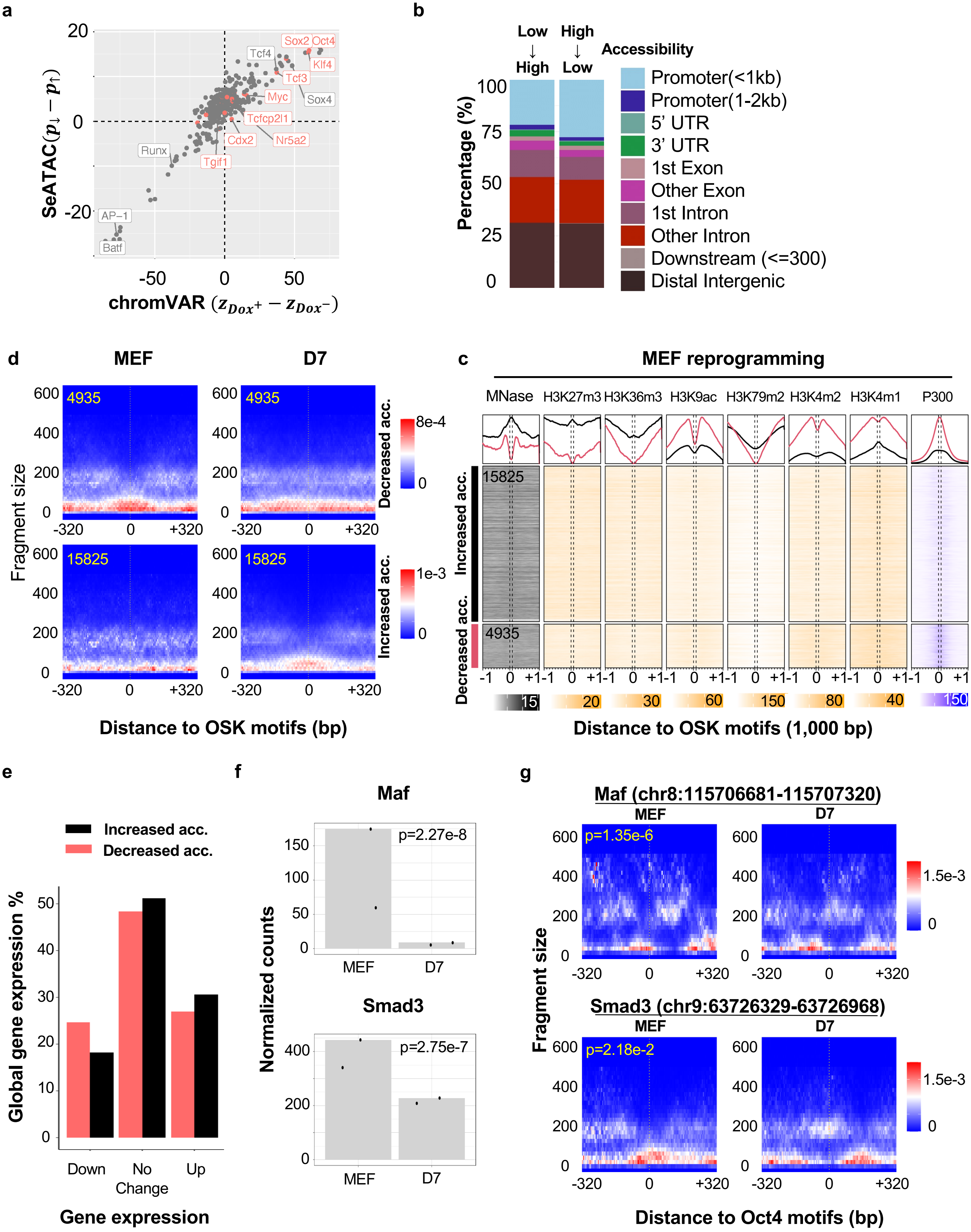
a
SeATAC($p_{\downarrow} - p_{\uparrow}$)
chromVAR ($z_{Dox^+} - z_{Dox^-}$)
Sox2 Oct4
Tcf4
Klf4
Tcf3
Myc
Sox4
Tcfcp2l1
Cdx2
Nr5a2
Runx
Tgif1
AP−1
Batf
b
Low ↓ High
High ↓ Low
Accessibility
Percentage (%)
Promoter(<1kb)
Promoter(1-2kb)
5' UTR
3' UTR
1st Exon
Other Exon
1st Intron
Other Intron
Downstream (<=300)
Distal Intergenic
d
MEF
D7
Fragment size
4935
15825
Decreased acc.
Increased acc.
8e-4
1e-3
Distance to OSK motifs (bp)
c
MEF reprogramming
MNase
H3K27m3
H3K36m3
H3K9ac
H3K79m2
H3K4m2
H3K4m1
P300
Increased acc.
Decreased acc.
Distance to OSK motifs (1,000 bp)
e
Increased acc.
Decreased acc.
Global gene expression %
Down
No Change
Up
Gene expression
f
Maf
p=2.27e-8
Normalized counts
MEF
D7
Smad3
p=2.75e-7
g
Maf (chr8:115706681-115707320)
MEF
D7
p=1.35e-6
1.5e-3
Smad3 (chr9:63726329-63726968)
p=2.18e-2
Fragment size
Distance to Oct4 motifs (bp)

Fig. S10. Induction of OSK (Oct4/Sox2/Klf4) causes both chromatin relaxation and closure at OSK binding sites. (a) The dot plots compare the changes of motif associated chromatin accessibility estimated by chromVAR (x-axis) and the difference of the percent of TFBS with decreased or increased chromatin accessibility estimated by SeATAC (y-axis). $\boldsymbol{z_{Dox^+}}$ and $\boldsymbol{z_{Dox^-}}$ are the normalized deviation score of Dox^+ condition (7 days post-induction for MEF reprogramming) and Dox^- condition (undifferentiated MEFs). $\boldsymbol{p_{\downarrow}}$ and $\boldsymbol{p_{\uparrow}}$ are the percent of TFBS that shows decreased or increased chromatin accessibility in Dox^+ condition compared with the Dox^- condition. **(b)** The barplots show the genomic distribution of OSK binding sites with decreased (NFR->NOR) or increased (NOR->NFR) chromatin accessibility in MEF reprogramming. The change of chromatin accessibility is estimated using SeATAC. **(c)** The aggregated V-plot include 15,825 and 4,935 OSK binding sites that have increased (NOR->NFR) or decreased (NFR->NOR) chromatin accessibility during MEF reprograming. The heatmap color indicates the estimated read density. **(d)** The heatmaps show the MNase-seq, H3K27m3, H3K36m3, H3K9ac, H3K79me2, H3K4me2, H3K4me1 and P300 ChIP-seq signals in undifferentiated MEFs of 15,825 and 4,935 OSK binding sites that have increased (NOR->NFR) or decreased (NFR->NOR) chromatin accessibility during the MEF reprogramming. The change of chromatin accessibility is estimated using SeATAC. **(e)** The barplots show the percent of genes that were down-regulated, up-regulated or not changed between undifferentiated MEFs and 7 hours post OSK induction. **(f-g)** Maf and Smad3 **(f)** are significantly down-regulated during the OSK induced MEF reprogramming and **(g)** have OSK motifs that become significantly less accessible during the differentiation at their promoter region (-5,000 - +1,000bp region flanking the TSS). The heatmap color indicates estimated read density.

Fig. S11

a

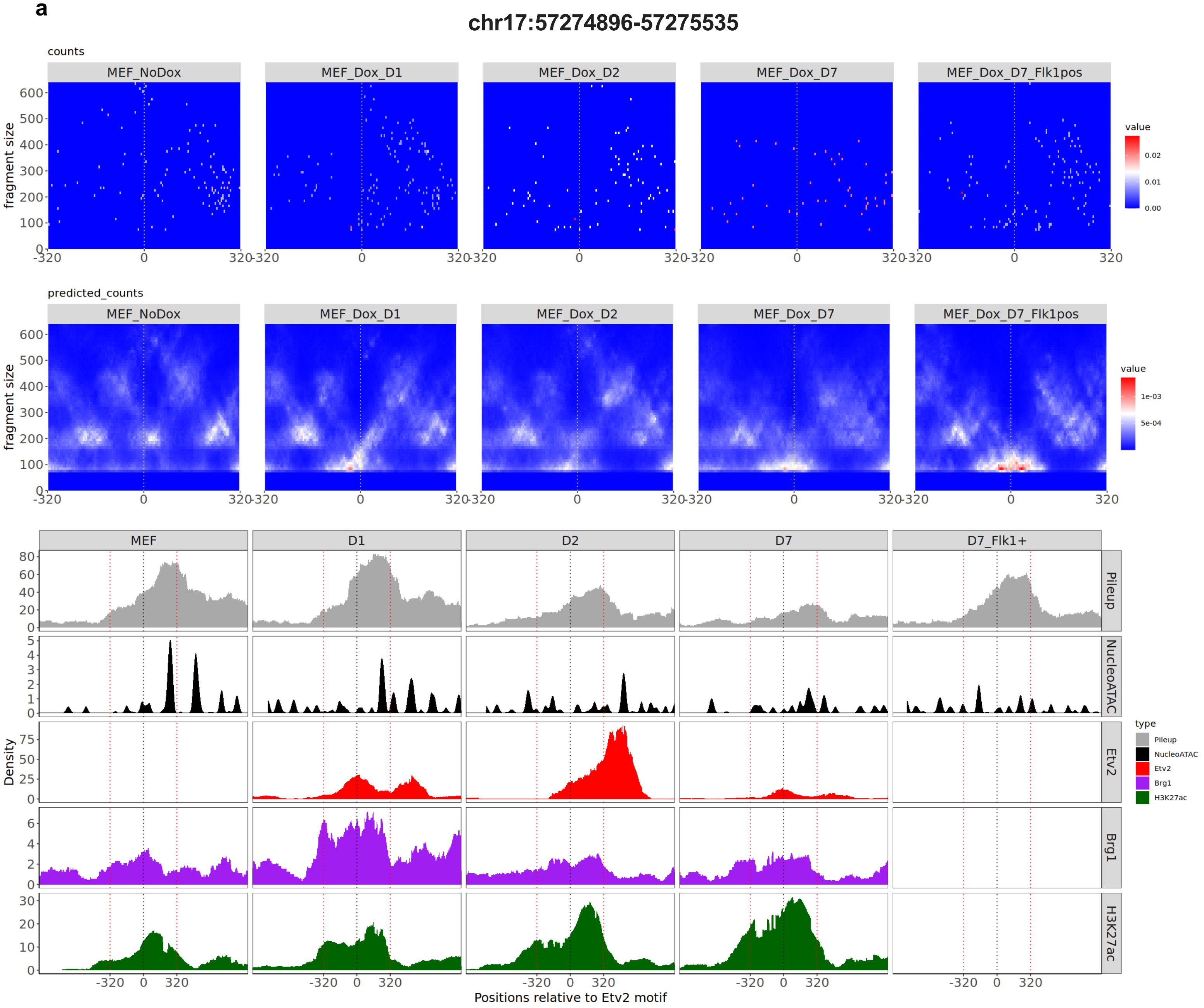

chr17:57274896-57275535
counts
MEF_NoDox
MEF_Dox_D1
MEF_Dox_D2
MEF_Dox_D7
MEF_Dox_D7_Flk1pos
fragment size
value
predicted_counts
MEF
D1
D2
D7
D7_Flk1+
Pileup
NucleoATAC
Etv2
Brg1
H3K27ac
Density
type
Positions relative to Etv2 motif

Fig. S11

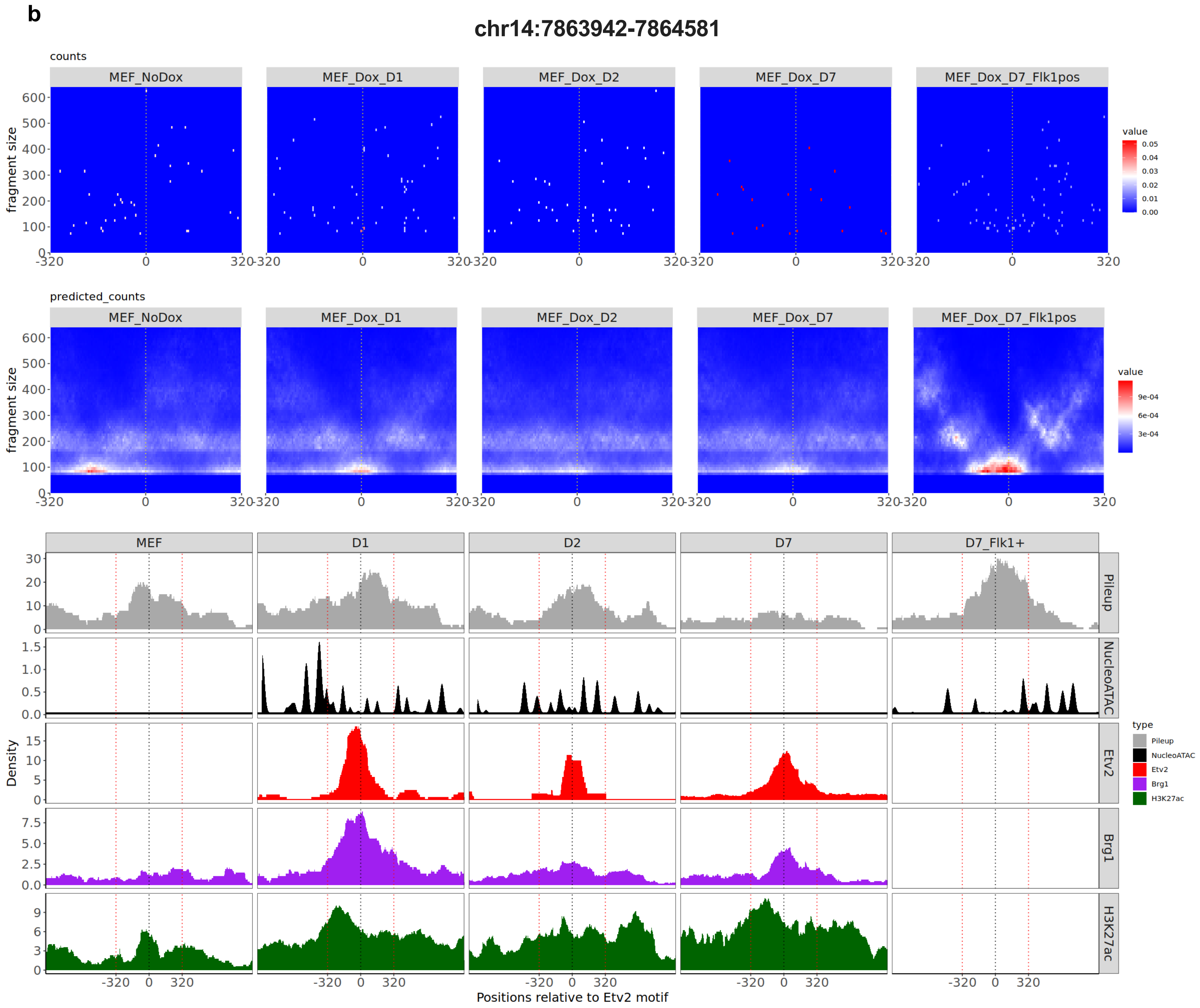
b
chr14:7863942-7864581
counts
MEF_NoDox
MEF_Dox_D1
MEF_Dox_D2
MEF_Dox_D7
MEF_Dox_D7_Flk1pos
fragment size
value
predicted_counts
MEF
D1
D2
D7
D7_Flk1+
Pileup
NucleoATAC
Etv2
Brg1
H3K27ac
Density
type
Positions relative to Etv2 motif

Fig. S11

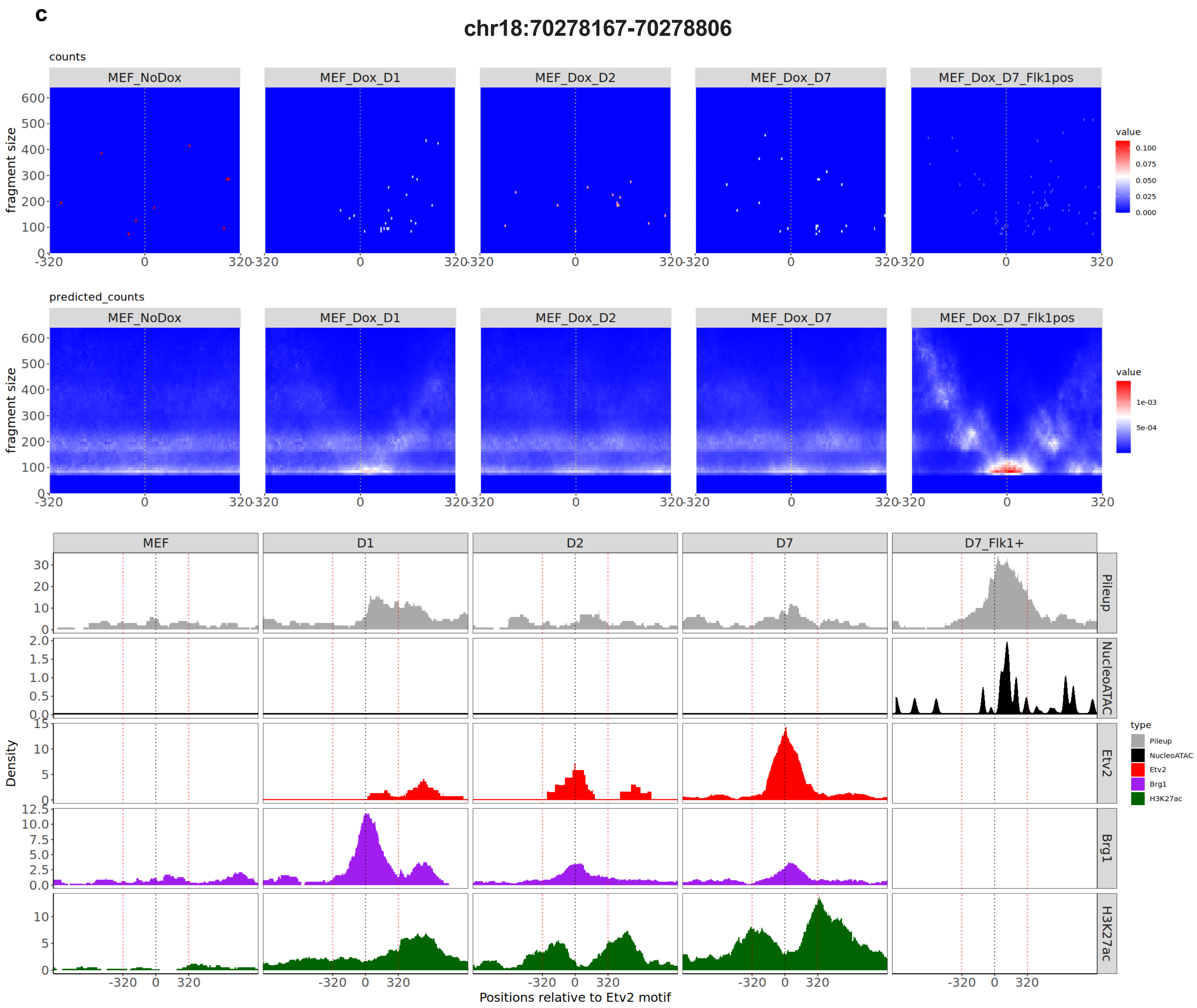

c
chr18:70278167-70278806
counts
MEF_NoDox
MEF_Dox_D1
MEF_Dox_D2
MEF_Dox_D7
MEF_Dox_D7_Flk1pos
fragment size
value
predicted_counts
MEF
D1
D2
D7
D7_Flk1+
Pileup
NucleoATAC
Etv2
Brg1
H3K27ac
Density
type
Positions relative to Etv2 motif

Fig. S11

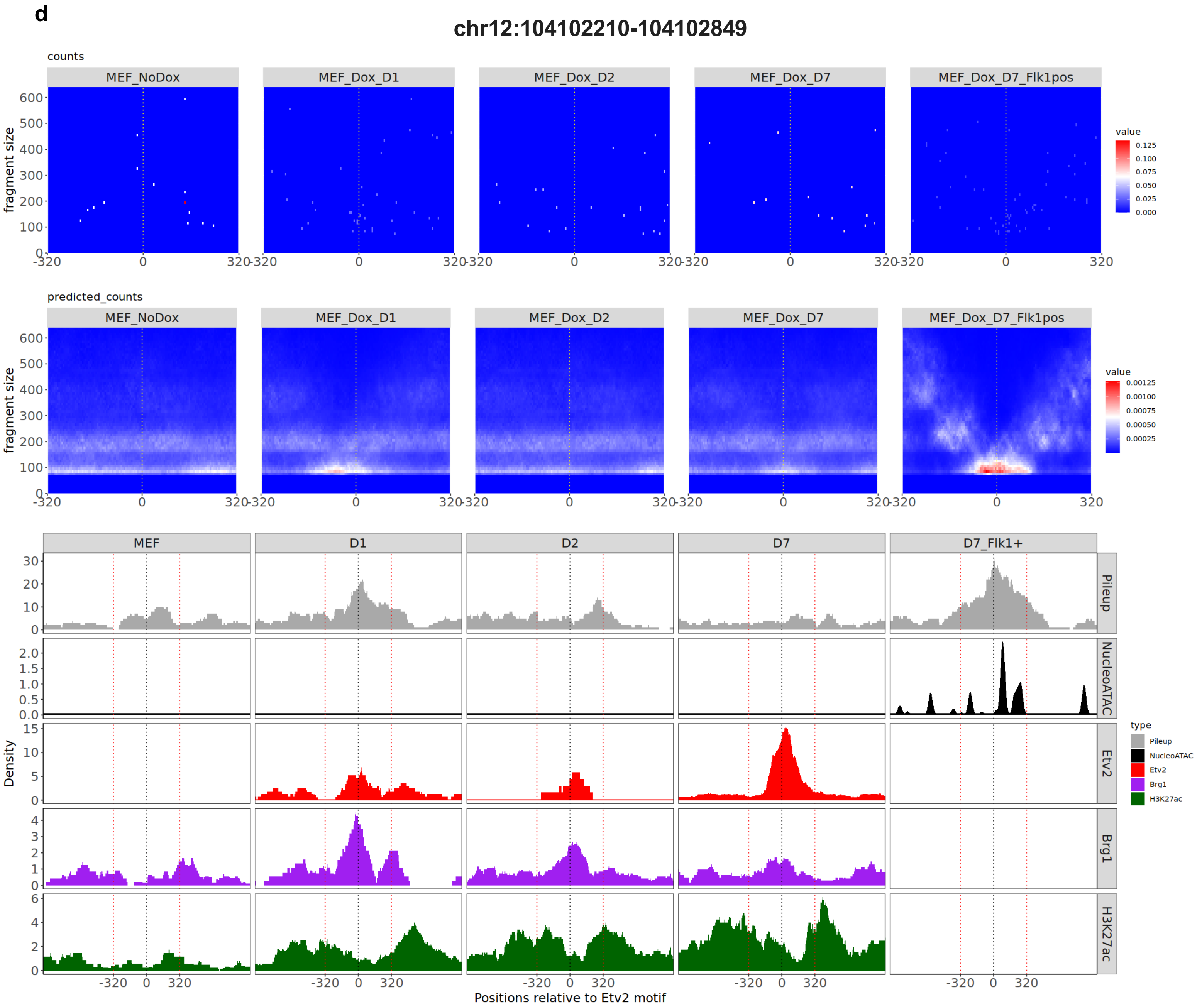

d
chr12:104102210-104102849
counts
MEF_NoDox
MEF_Dox_D1
MEF_Dox_D2
MEF_Dox_D7
MEF_Dox_D7_Flk1pos
fragment size
value
predicted_counts
MEF
D1
D2
D7
D7_Flk1+
Pileup
NucleoATAC
Etv2
Brg1
H3K27ac
Density
type
Positions relative to Etv2 motif

Fig. S11

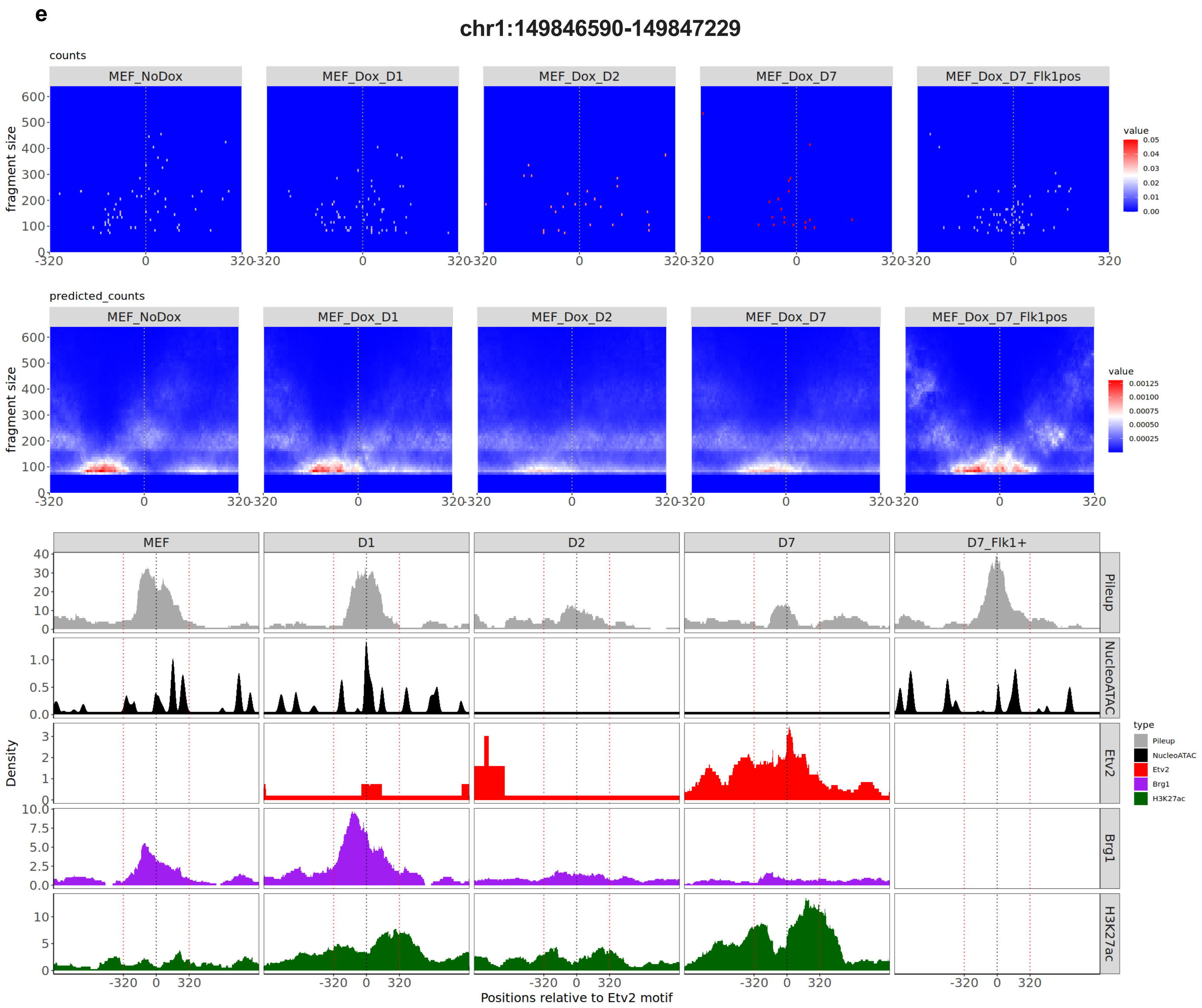
e
chr1:149846590-149847229
counts
MEF_NoDox
MEF_Dox_D1
MEF_Dox_D2
MEF_Dox_D7
MEF_Dox_D7_Flk1pos
fragment size
value
predicted_counts
MEF
D1
D2
D7
D7_Flk1+
Pileup
NucleoATAC
Etv2
Brg1
H3K27ac
Density
type
Positions relative to Etv2 motif

Fig. S11

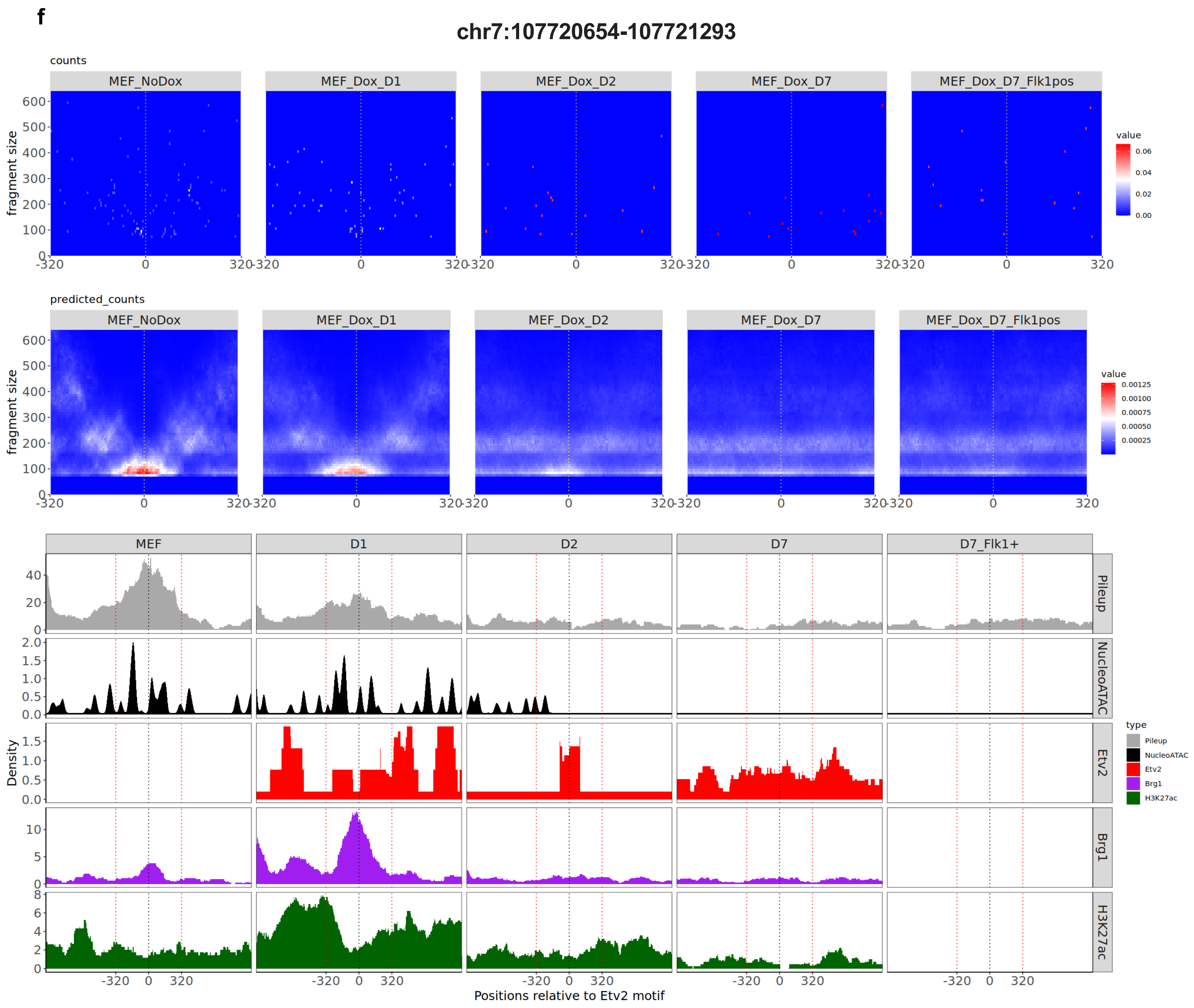
f
chr7:107720654-107721293
counts
MEF_NoDox
MEF_Dox_D1
MEF_Dox_D2
MEF_Dox_D7
MEF_Dox_D7_Flk1pos
fragment size
value
predicted_counts
MEF
D1
D2
D7
D7_Flk1+
Pileup
NucleoATAC
Etv2
Brg1
H3K27ac
Density
type
Positions relative to Etv2 motif

Fig. S11

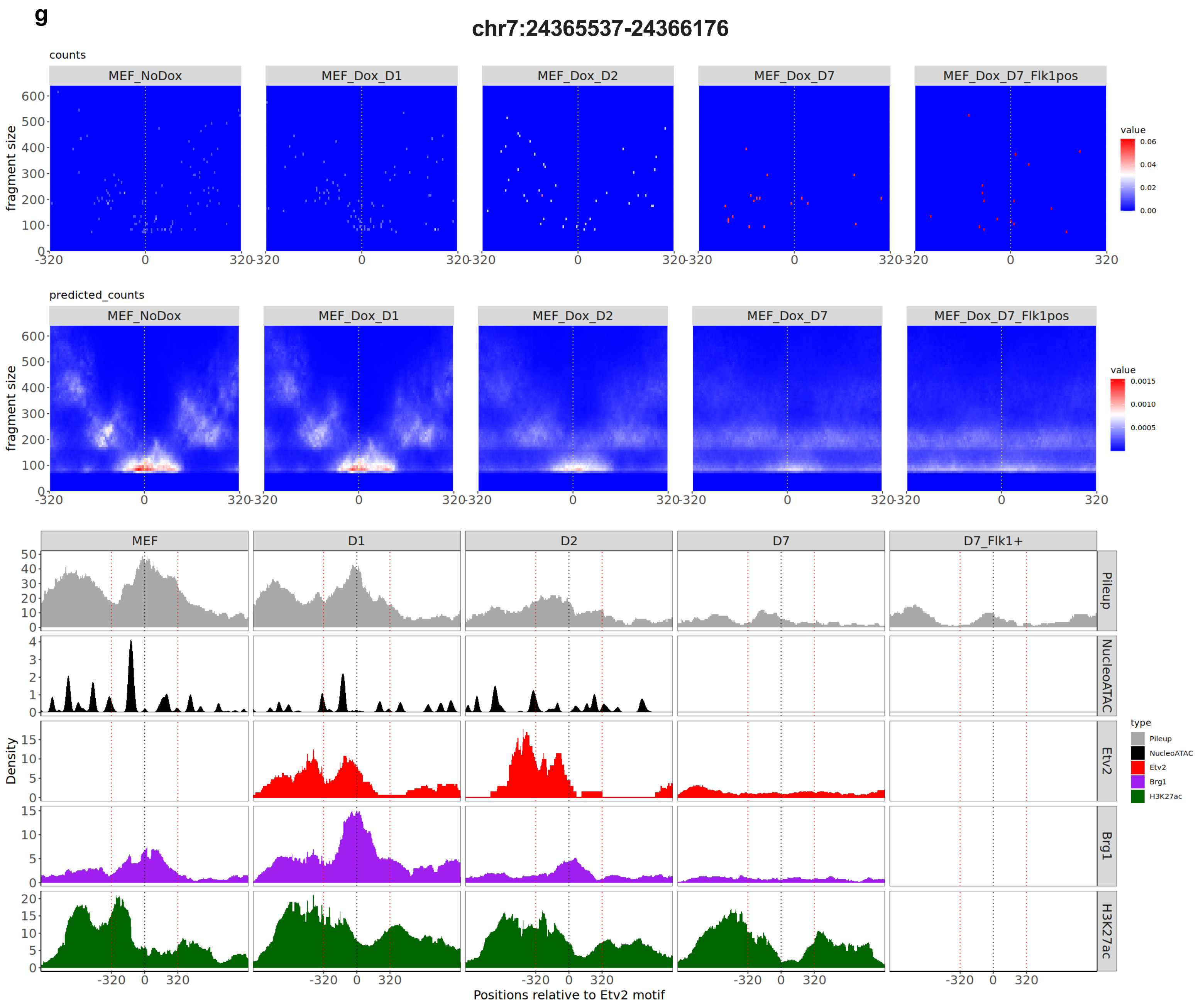

g
chr7:24365537-24366176
counts
MEF_NoDox
MEF_Dox_D1
MEF_Dox_D2
MEF_Dox_D7
MEF_Dox_D7_Flk1pos
fragment size
value
predicted_counts
MEF
D1
D2
D7
D7_Flk1+
Pileup
NucleoATAC
Etv2
Brg1
H3K27ac
Density
type
Positions relative to Etv2 motif

Fig. S11

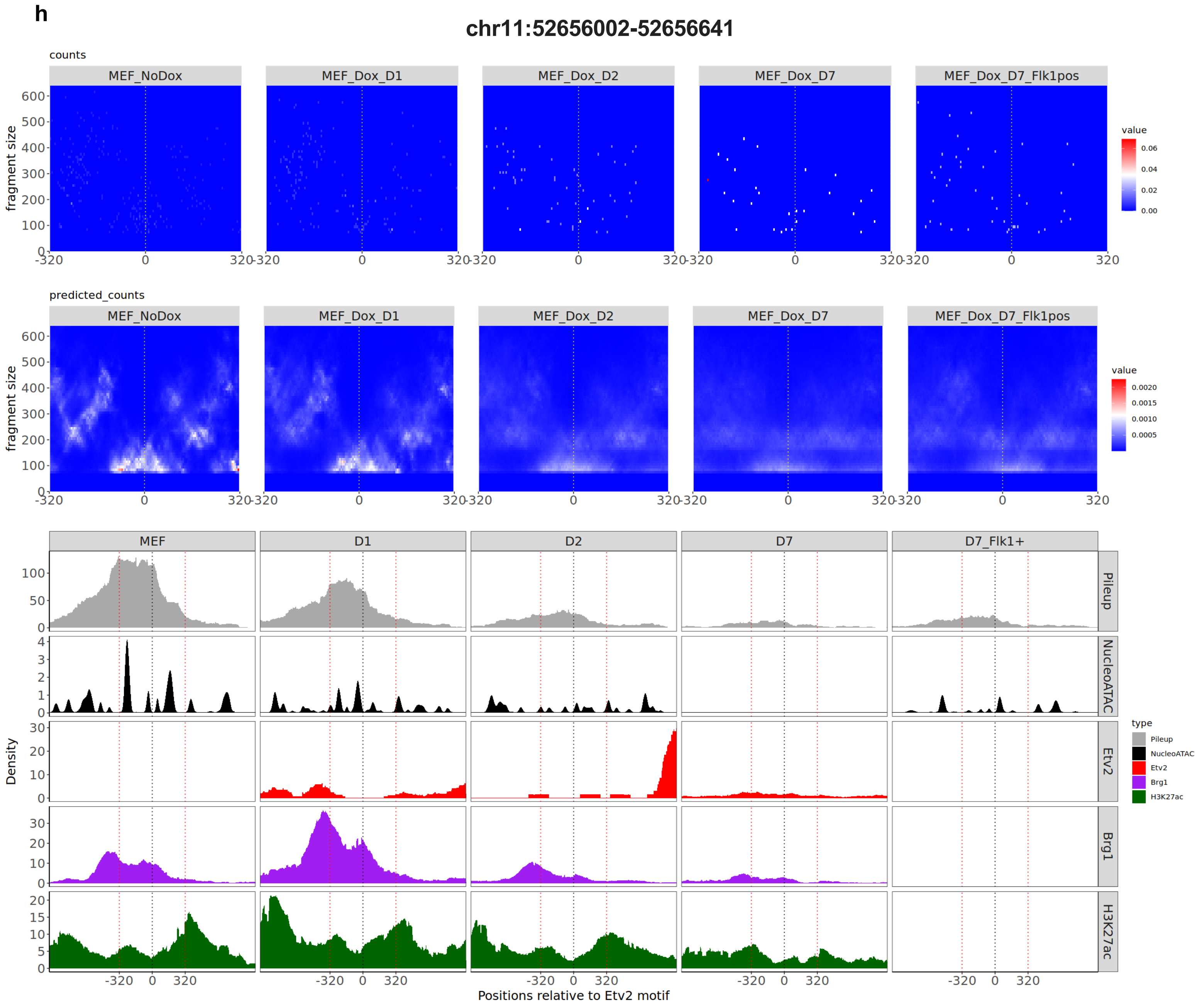

h
chr11:52656002-52656641
counts
MEF_NoDox
MEF_Dox_D1
MEF_Dox_D2
MEF_Dox_D7
MEF_Dox_D7_Flk1pos
fragment size
value
predicted_counts
MEF
D1
D2
D7
D7_Flk1+
Pileup
NucleoATAC
Etv2
Brg1
H3K27ac
Density
type
Positions relative to Etv2 motif

Fig. S11

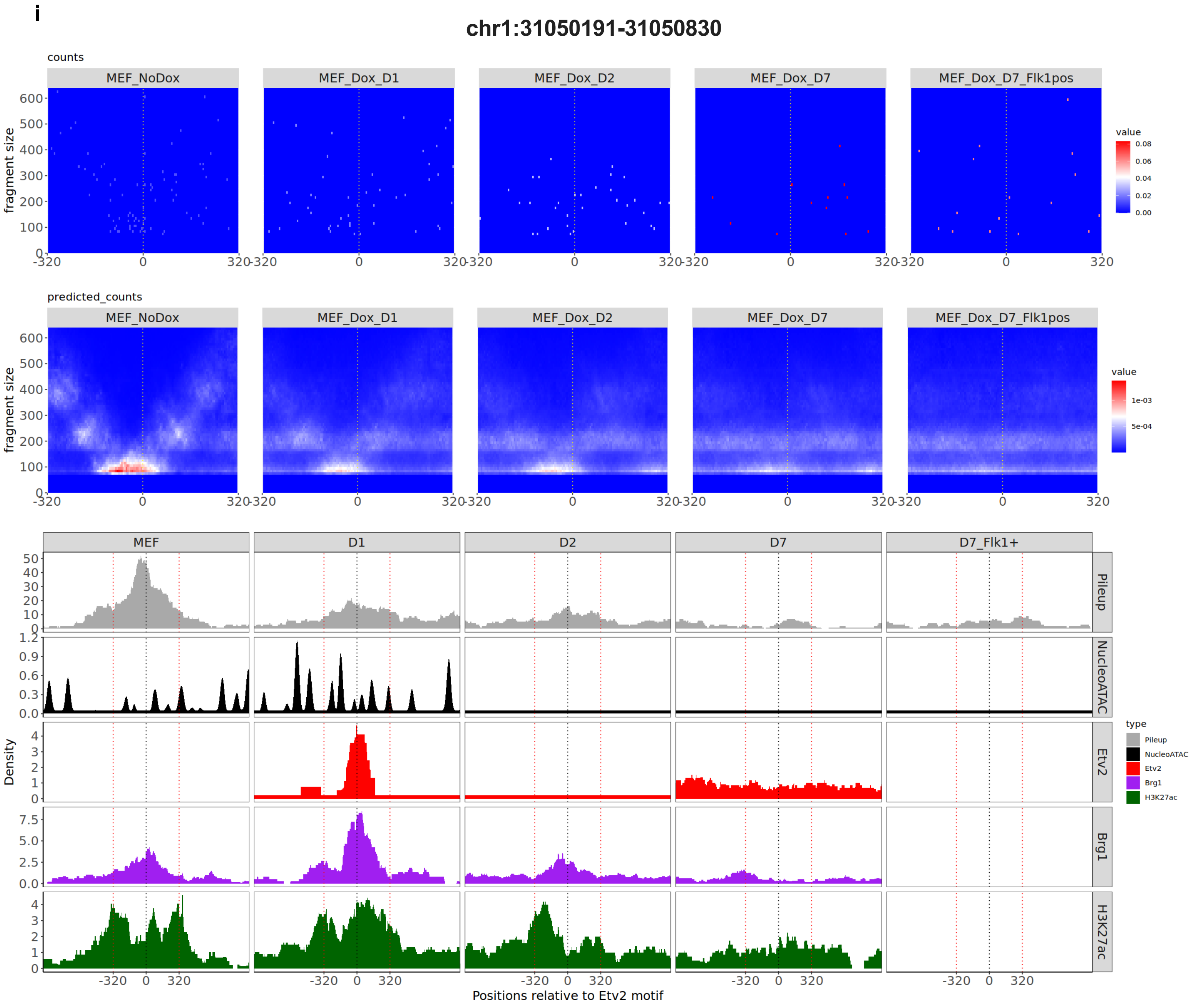
i
chr1:31050191-31050830
counts
MEF_NoDox
MEF_Dox_D1
MEF_Dox_D2
MEF_Dox_D7
MEF_Dox_D7_Flk1pos
fragment size
value
predicted_counts
MEF
D1
D2
D7
D7_Flk1+
Pileup
NucleoATAC
Etv2
Brg1
H3K27ac
Density
type
Positions relative to Etv2 motif

Fig. S11

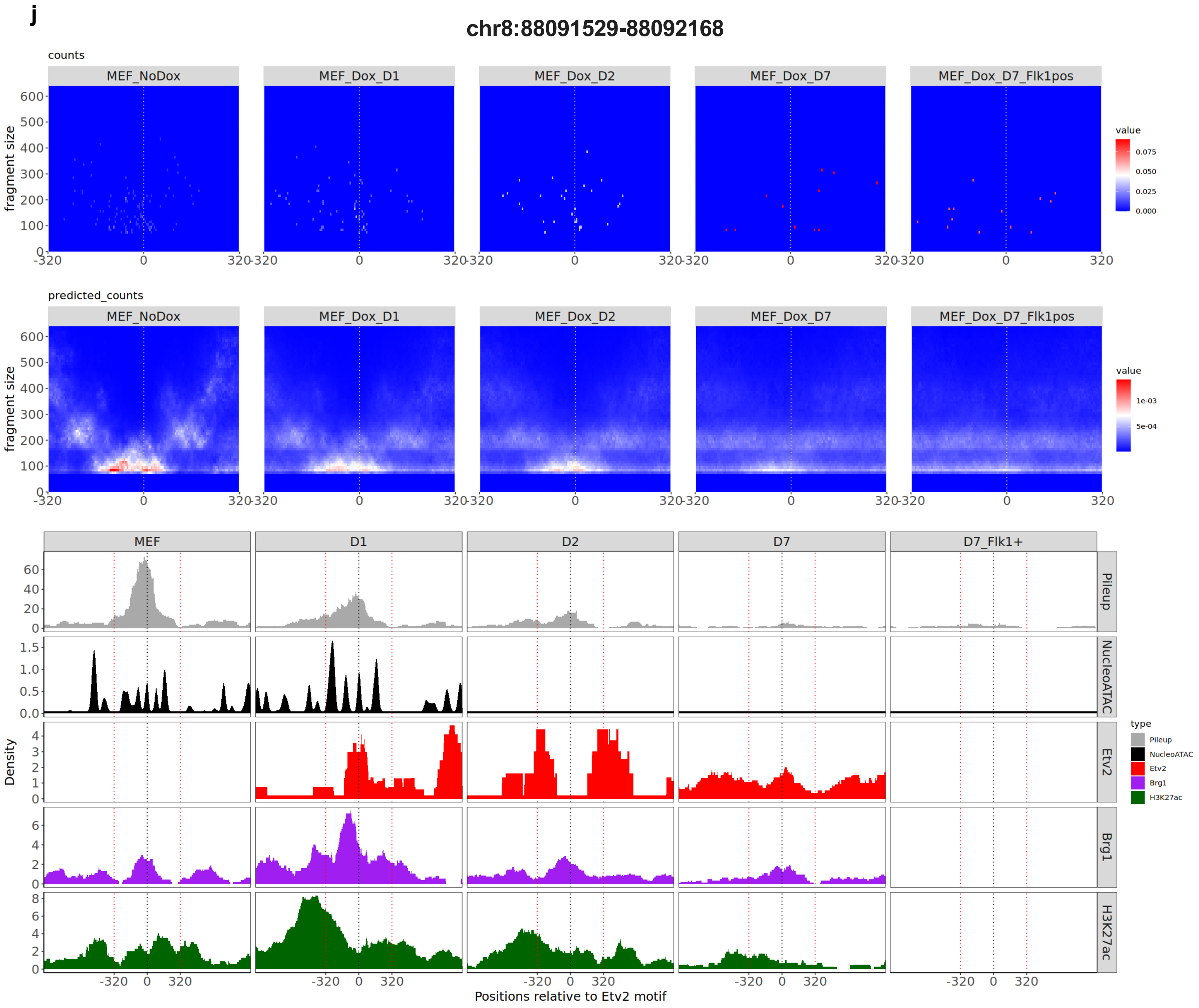

j
chr8:88091529-88092168
counts
MEF_NoDox
MEF_Dox_D1
MEF_Dox_D2
MEF_Dox_D7
MEF_Dox_D7_Flk1pos
fragment size
value
0.075
0.050
0.025
0.000
predicted_counts
1e-03
5e-04
MEF
D1
D2
D7
D7_Flk1+
Pileup
NucleoATAC
Etv2
Brg1
H3K27ac
Density
type
Positions relative to Etv2 motif

Fig. S11. SeATAC detects Etv2 binding sites with significantly altered chromatin accessibility during the Etv2 induced reprogramming. (a-e) Example regions with significantly increased chromatin accessibility from undifferentiated MEFs to D7 Flk1^{+} samples. **(f-j)** Example regions with significantly decreased chromatin accessibility from undifferentiated MEFs to D7 Flk1^{+} samples.
