# Supplementary material for: SeATAC: a tool for exploring the chromatin landscape and the role of pioneer factors
Source: Genome Biol. 2023 May 22;24:125. doi: 10.1186/s13059-023-02954-5 (PMC10204251; doi:10.1186/s13059-023-02954-5)
Supplement: Supplementary file 2 — Additional file 2. Review history. [file 13059_2023_2954_MOESM2_ESM.docx]

Review History

**First round of review**

**Reviewer 1**

**Were you able to assess all statistics in the manuscript, including the appropriateness of statistical tests used?**

Yes, and I have assessed the statistics in my report.

**Comments to author:**

Deep learning has been widely recognized for its nonlinear representation for unstructured and noisy data in bio-medicine studies. In this paper, the authors developed a bioinformatics tool called SeATAC. They mainly used a conditional variational autoencoder (CVAE) model to learn the latent representation of ATAC-seq V-plots, and to estimate the statistically differential chromatin accessibility. They compared SeATAC with existing methodds MACS2 and NucleoATAC on four separate tasks including: (1) detection of differential V-plots; (2) definition of nucleosome positions; (3) detection of nucleosome changes and (4) designation of transcriptional factor binding sites (TFBS) with differential chromatin accessibility. Specifically they applied SeATAC to several pioneer factor induced differentiation or reprogramming ATAC-seq datasets and provides some biological insights on these pioneer factors associated with different genomic distribution and histone marks. Overall, the authors leveraged the power of deep learning in image data and presented SeATAC as a novel tool to accurately reveal the genomic regions with differential chromatin accessibility from ATAC-seq data.

My major concern is from the method validation. Currently SeATAC was mainly evaluated on the datasets down-sampled from a full ATAC-seq dataset. Since the Vplot is very sparse and noise data, it's very difficult to tell if the results are indeed biologically meaningful purely relying on ATAC-seq data itself. Independent data will be helpful in this case. Especially downstream gene expression data will provide key information for the chromatin state. There are many paired expression and chromatin accessibility data from ENCODE and ROADMAP. As demonstrated in the following reference, integrating two data layer provides better gene expression prediction. The author should consider to systematically evaluate the method with gene expression data.

Modeling gene regulation from paired expression and chromatin accessibility data. Proceedings of the National Academy of Sciences 114 (25), E4914-E4923

Instead of the ROC curve to show the overall accuracy for SeATAC, it will be great to show some examples, in which SeATAC outperform the existing methods. It's better to point out how deep learning can capture the subtle Vplot patterns.

How the SeATAC method depended on the ATAC-seq data quality? There are many ATAC-seq available with different sequencing depth and many quality control indexes. The authors should systematically investigate how the SeATAC output is correlated with the data quality. Ideally, the authors should provide some guidelines for when to use SeATAC and the suggestion for data quality index for better nucleosome position study. Or how many read counts in one Vplot is needed for SeATAC? How is the requirement in existing methods?


The pioneer factor study is interesting. Since there are no clear definition for pioneer TF. Those subtle pattern in SeATAC should be helpful. Again this part should be compared with paired gene expression and accessibility analysis (MEF in the following ref.). Also some concrete examples will be helpful than the overall picture.

Time course regulatory analysis based on paired expression and chromatin accessibility data. Genome research 30 (4), 622-634

**Reviewer 2**

**Were you able to assess all statistics in the manuscript, including the appropriateness of statistical tests used?**

No, I do not feel adequately qualified to assess the statistics.

**Comments to author:**

This paper describes a novel algorithm for the differential analyses of ATAC-seq profiles. The method is validated using various datasets from GEO relating to ATAC-seq profiles before and after the action of pioneer transcription factors. Validation of improvements with the SeATAC tool is mostly documented through detection of larger sets of differentially accessible regions. Further, these enhanced subsets are validated through Gene Ontology analyses for perceived biological relevance.

This reviewer is a biologist, not a bioinformatician, and accordingly my comments originate from that viewpoint. The SeATAC tool appears to be an improvement compared to currently available methods, but the validation of the improvements provided by SeATAC is difficult to appraise from the bulk numbers provided. Indeed, it is not the number of sites but rather their biological relevance that needs to be conveyed; the GO analyses go only some way towards validation. The paper would be far more useful/convincing for biologists if it included a series of browser views for the sites that are detected with seATAC but not with other procedures in order to inform on the validity of the interpretations. Further, as SeATAC appears to be unique in documenting decreased chromatin accessibility at promoters of regulated genes, this aspect would be particularly interesting to biologists and browser views would be very helpful to convince the readers. To be really helpful, these browser views should include other typical marks of promoters and enhancers such as H3K4me3, H3K4me1, H3K27ac, etc. Finally, an attempt should be made to define the limitations of SeATAC, for example by comparisons with sites of biological relevance identified/discussed in the original papers that reported the datasets.

The terminology used to characterize the accessible domains identified by SeATAC is a bit simplistic, if not shocking, because it only offers a black and white, positive and negative, view of situations. For example, referring to NOR, nucleosome occupied regions, for promoters that become less accessible following the action of pioneers is inappropriate because these promoters show decrease in accessibility but do not become "fully occupied" as suggested by the wording. Since the ATAC measurements record DNA accessibility, it would be more appropriate to use acronyms that refers to DNA accessibility rather than to nucleosome occupied or free regions: the latter is a gross overinterpretation. Referring to increased or decreased accessibility would be more appropriate.

Minor comments
L.65 Protected or unprotected?
L.155 "Regarding".

**Authors Response**

**Point-by-point responses to the reviewers’ comments:**

Thank you for the critical and helpful comments regarding our manuscript (GBIO-D-22-00608), entitled " SeATAC: a tool for exploring the chromatin landscape and the role of pioneer factors". We have examined the comments carefully and performed a number of additional analyses as requested by the reviewers and these are now included in our revised manuscript. These new analyses/studies include the following:

1. Expanded the benchmark analysis (task #1 - #3) on 523 ATAC-seq samples from 20 published studies
2. Examined the performance of SeATAC on eight ATAC-seq quality indices
3. Evaluated the performance of predicting differentially expressed genes from the ATAC-seq signals over their transcriptional start sites (TSS) on 17 paired RNA-seq / ATAC-seq datasets
4. Evaluated the performance of predicting histone modification signals (H3K27ac, H3K4me1 and h3K4me3) from chromatin accessibility representations produced by SeATAC, NucleoATAC and MACS2
5. Provided concrete examples of pioneer factor induced changes of chromatin accessibility

New data are presented in 34 panels which include: Fig. 2c- 2e, Fig. 3c-3d, Fig. 4d-4e, Supplementary Figure 2a, Supplementary Figure 3a-3h, Supplementary Figure 4a-4c, Supplementary 5a-5d, Supplementary Figure 11a-11j and one new Table. The new data were generated from the analyses outlined above and as requested by the reviewers. All of the new results support our original conclusions, and we believe that the manuscript has been significantly strengthened by these additional data. As outlined below, we provide point-by-point responses to the reviewers' comments and highlight the additional analysis that have been performed. Each issue raised by the reviewer is provided as a direct quote and is followed by our response. These additional studies provide new information that will be available to the field, and we hope that the reviewers will find the manuscript suitable for publication. The code to reproduce main figures and supplementary figures can be found at the GitHub site: https://github.com/gongx030/seatac_manuscript.


**Reviewer #1:** “Deep learning has been widely recognized for its nonlinear representation for unstructured and noisy data in bio-medicine studies. In this paper, the authors developed a bioinformatics tool called SeATAC. They mainly used a conditional variational autoencoder (CVAE) model to learn the latent representation of ATAC-seq V-plots, and to estimate the statistically differential chromatin accessibility. They compared SeATAC with existing methods MACS2 and NucleoATAC on four separate tasks including: (1) detection of differential V-plots; (2) definition of nucleosome positions; (3) detection of nucleosome changes and (4) designation of transcriptional factor binding sites (TFBS) with differential chromatin accessibility. Specifically, they applied SeATAC to several pioneer factor induced differentiation or reprogramming ATAC-seq datasets and provides some biological insights on these pioneer factors associated with different genomic distribution and histone marks. Overall, the authors leveraged the power of deep learning in image data and presented SeATAC as a novel tool to accurately reveal the genomic regions with differential chromatin accessibility from ATAC-seq data.

Issue #1. My major concern is from the method validation. Currently SeATAC was mainly evaluated on the datasets down-sampled from a full ATAC-seq dataset. Since the Vplot is very sparse and noise data, it's very difficult to tell if the results are indeed biologically meaningful purely relying on ATAC-seq data itself. Independent data will be helpful in this case. Especially downstream gene expression data will provide key information for the chromatin state. There are many paired expression and chromatin accessibility data from ENCODE and ROADMAP. As demonstrated in the following reference, integrating two data layer provides better gene expression prediction. The author should consider to systematically evaluate the method with gene expression data.

Modeling gene regulation from paired expression and chromatin accessibility data. Proceedings of the National Academy of Sciences 114 (25), E4914-E4923

*Response: We thank and agree with Reviewer #1 regarding their comments. In the revised manuscript, we included two additional analyses to evaluate the performance of SeATAC on predicting (1) differentially expressed genes (the new task #4), and (2) histone modifications (the new task #5), respectively. Both two tasks were designed to evaluate how accurate the local ATAC-seq information captured by SeATAC, NucleoATAC or MACS2 was able to predict the biologically relevant readout such as differentially expressed genes or local histone modification.

We agree with Reviewer #1 that modeling and integrating RNA-seq and ATAC-seq at a global level could provide more accurate gene expression prediction and infer the regulatory elements [1,2]. SeATAC was designed as a tool to model the local ATAC-seq data as a V-plot and to provide more accurate information regarding the local chromatin accessibility changes, such as nucleosome positions and nucleosome phasing [3,4], while modeling the relationship between any local ATAC-seq signal and global gene expression levels was beyond the scope of SeATAC. Previous studies have shown that the changes of DNA accessibility over the promoter regions were weakly associated with the gene expression changes [5,6]. However, these studies used the simple ATAC-seq peaks or density as the features for correlating with the RNA-seq levels. We asked whether including more sophisticated ATAC-seq features such as V-plot would improve the accuracy of predicting gene expression changes.

We compiled a list of 17 paired RNA-seq / ATAC-seq datasets on temporal reprogramming or cellular differentiation (Table 1). In each dataset, we first compared the RNA-seq of data from any two conditions (e.g. treatment vs control) and determined a list of significantly up- and down-regulated genes (DESeq2 q-value<0.05 with >2 fold change)[7]. Then we used SeATAC, NucleoATAC and MACS2 to generate features from the ATAC-seq signals over the promoter regions of the significantly differentially expressed (DE) genes. For SeATAC, the latent representations of the V-plots centering at transcription start sites (TSS) were used. We used principal component analysis (PCA) of the differential pileup signals (MACS2's pileup output) and the differential nucleosome signals (NucleoATAC's smooth signal output) to construct the features for MACS2 and NucleoATAC, respectively. We kept the feature dimensions the same for the three tools. Next, we built simple logistical regression models to predict the up- or down-regulated genes (a classification task) from the corresponding features and used the 5-fold cross-validation (CV) to evaluate the prediction (Supplementary Figure 4a). We found that the feature dimensions had moderate impact on the prediction performance of MACS2 and increasing the latent dimension from 10 to 20 significantly improved the performance of SeATAC (Wilcoxson rank-sum test p-value < 0.05), while increasing the promoter regions (e.g. from 640 bp to 2560 bp) did not improve the performance of SeATAC (Supplementary Figure 4b). Because of the difference in the experimental setting and the data quality, the absolute AUC (area under ROC) varied significantly over 17 examined datasets. For example, SeATAC had relatively more accurate prediction of DE genes from Ascl1 reprogramming dataset (mean AUC=0.754) [8,9]. Overall, we found that SeATAC had the best performance of predicting DE genes over 16 out of 17 datasets, suggesting that the V-plot representations produced by SeATAC better captured the relationship between DNA accessibility at the promoter and the gene expression changes (Supplementary Figure 4c).

For evaluating the performance of predicting histone modifications from the V-plot representations produced by SeATAC, please see our response to Reviewer #2's Issue #2.*

Issue #2. Instead of the ROC curve to show the overall accuracy for SeATAC, it will be great to show some examples, in which SeATAC outperform the existing methods. It's better to point out how deep learning can capture the subtle V-plot patterns.

*Response: We provided concrete examples of Etv2 binding sites with significantly altered chromatin accessibility during the Etv2 induced reprogramming in Supplementary Figure 11 [3]. This dataset includes ATAC-seq data of undifferentiated MEFs, day 1 (D1), day 2 (D2), day 7 (D7) and Flk1+ cells from day 7 (D7 Flk+) post Etv2 induction. Supplementary Figure 11a-11e showed five instances of Etv2 binding sites with significantly increased chromatin accessibility from MEFs to day 7 Flk1+ samples, as detected by SeATAC (adjusted p-value < 0.05 and δ^NOR<-0.2). In contrast, NucleoATAC did not show contrastive nucleosome signals at the center of these binding sites between MEFs and D7 Flk+ samples. As we can see from these examples, as well as the global pattern of 3,996 Etv2 binding sites with increased chromatin accessibility (Supplementary Figure 6b), Etv2 binding associated increasing accessibility was coupled with increased nearby Brg1 signal, H3K27ac signal, and a "dip" in the H3K27ac peak. The establishment of H3K27ac modification surrounding the pioneer factor bound sites was also found in ASCL1 driven neural differentiation and OSKM driven MEF reprogramming [3,10,11].

On the other hand, Supplementary Figures 11f-11j showed five instances of Etv2 binding sites with significantly decreased chromatin accessibility from MEFs to day 7 Flk1+ samples (adjusted p-value < 0.05 and δ^NOR>0.2). NucleoATAC did not produce contrastive nucleosome signals at the Etv2 binding site between MEFs and D7 Flk+ samples. The decrease of chromatin accessibility was also coupled with the decrease of Brg1 (SMARCA4) density, a key SWI/SNF-related chromatin-remodeling complex that facilitates chromatin relaxation[12].

We believe that these examples provide detailed interpretation of how SeATAC estimated the V-plot from the raw ATAC-seq reads and discovered the changes of chromatin accessibility.*

Issue #3. How the SeATAC method depended on the ATAC-seq data quality? There are many ATAC-seq available with different sequencing depth and many quality control indexes. The authors should systematically investigate how the SeATAC output is correlated with the data quality. Ideally, the authors should provide some guidelines for when to use SeATAC and the suggestion for data quality index for better nucleosome position study. Or how many read counts in one Vplot is needed for SeATAC? How is the requirement in existing methods?

*Response: To systematically and comprehensively evaluate the performance of SeATAC, we performed three separate tasks: (1) detection of differential V-plot, (2) definition of nucleosome positions and (3) detection of nucleosome changes, as well as the performance of SeATAC on different ATAC-seq quality indices. We extended the down-sampling analysis based on only GM12878 ATAC-seq data [13] to 523 ATAC-seq samples from 20 published studies [1,8,14–32]. We used SeATAC, NucleoATAC and MACS2 to perform three tasks on each of 523 ATAC-seq samples, according to the same procedure described in the Methods section. This systematic analysis confirmed the superior performance of SeATAC over the other tools on (1) detection of differential V-plot (Figure 2d), (2) definition of nucleosome positions (Figure 3c), and (3) detection of nucleosome changes (Figure 4d), where the AUC (area under ROC) of SeATAC was significantly higher than the other tools in all three tasks (Wilcoxon rank sum test p-value < 1e-10).

To further elaborate the performance of SeATAC on other parameters, as Reviewer #1 suggested, we investigated the relationship between ATAC-seq read counts of a V-plot / genomic region and the performance of three tools. We found that for the detection of differential V-plots (task #1), the performance of SeATAC was not correlated with the read count cutoff (Figure 2e), while for the definition of nucleosome positions (task #2) and detection of nucleosome changes (task #3), SeATAC had better performance with increased number of ATAC-seq reads in the V-plot (Figure 3d and 4e).

We also examined the relationship between the performance of SeATAC on 8 ATAC-seq quality indices including (1) total number of reads (Total QNAMEs), (2) mitochondria rate, (3) proper pair rate, (4) unmapped rate, (5) the rate of a read has unmapped mate (has unmapped mate rate), (6) non redundant fraction, (7) PCR bottleneck coefficient 1 and (8) PCR bottleneck coefficient 2 [33,34]. The performance on task #1 was not significantly correlated with any quality indices. The performance on task #2 was significantly positively correlated with the total number of reads (Supplementary Figure 3a), proper pair rate (Supplementary Figure 3c), and negatively correlated with mitochondria rate (Supplementary Figure 3b), the unmapped rate (Supplementary Figure 3d), has-unmapped mate rate (Supplementary Figure 3e) (t-test p-value < 0.05). The performance on task #3 was only moderately positively correlated with the mitochondria rate (t-test p-value=0.036) (Supplementary Figure 3b).

In summary, these results suggested that SeATAC had better performance on ATAC-seq data with higher sequencing depth (e.g., average number of reads per V-plot), lower mitochondria rate, higher proper pair rate and lower unmapped rate, especially for task #2 (definition of nucleosome positions). For the other two tasks (detection of differential V-plot and detection of nucleosome changes) which focused on evaluating the changes of chromatin accessibility, the performance of SeATAC only moderately correlated with the ATAC-seq quality indices, while SeATAC had a more significant advantage over other tools on down-sampled sparse datasets.*

Issue #4. The pioneer factor study is interesting. Since there are no clear definition for pioneer TF. Those subtle pattern in SeATAC should be helpful. Again, this part should be compared with paired gene expression and accessibility analysis (MEF in the following ref.). Also some concrete examples will be helpful than the overall picture.

Time course regulatory analysis based on paired expression and chromatin accessibility data. Genome research 30 (4), 622-634

*Response: It has been shown by several studies that during development, pioneer transcription factors have the essential and unique role of opening new regulatory chromatin landscapes on genomic DNA [35]. The previous consensus is that pioneer factors can recognize their target DNA sequences in compacted chromatin, recruit chromatin remodelers and trigger the relaxation of the adjacent chromatin landscape to provide accessibility to non-pioneer transcription factors [36,37]. Several pioneer factors have been defined and all these factors have been shown to have important roles in various biological processes. Examples of pioneer factors include the pluripotent factors OSK (OCT4, SOX2, and KLF4) for iPSC reprogramming, FOXA for hepatocyte reprogramming[38], PAX7 for pituitary development[39], ASCL1 for neurogenesis[9] and ETV2 for endothelial reprogramming [3,4].

However, due to the lack of proper tools for quantitively dissecting nucleosomal changes of individual genomic regions (with relatively sparse ATAC-seq reads per region), previous studies have been largely focused on the aggregated genome-wise pattern. An important example is the overall nucleosome phasing events over all ASCL1 motifs during the ASCL1 induced neural reprogramming [8]. SeATAC provides a unique opportunity for researchers to examine individual genomic regions, due to its superior performance of detecting nucleosome changes on sparse data as demonstrated in our down-sampling analysis (Figure 4d and 4e). Besides the consensus opinion that pioneer factors can target the nucleosome and relax the closed chromatin, SeATAC detected the genomic regions where the pioneer factors could target the open chromatin and decrease the chromatin accessibility in various contexts, such as Etv2 induced EB differentiation (Figure 6d-6g), Ascl1 induced neural reprogramming (Supplementary Figure 9c-9e), OSKM induced reprogramming (Supplementary Figure 10c-10g) and Etv2 induced MEF reprogramming (Supplementary Figure 11a-11j). Additionally, we provided concrete examples of the Etv2 binding sites that were opened or closed by Etv2 binding during the Etv2 induced MEF reprogramming (Supplementary Figure 11a-11j). Please see our response to Reviewer #1's Issue #2).

As Reviewer #1 suggested, we compiled 17 paired temporal RNA-seq / ATAC-seq datasets on cellular differentiation or reprogramming and demonstrated that SeATAC had the best performance of predicting DE genes in 16 out of 17 datasets, suggesting that the V-plot representations produced by SeATAC better captured the relationship between DNA accessibility at the promoter and the gene expression changes (Supplementary Figure 4c). However, SeATAC focused on evaluating the changes of local chromatin accessibility, rather than modeling the global relationship between regulatory regions and changes of gene expression (e.g., PECA)[2]. Further studies are needed to dissect the mechanism that how decreasing chromatin accessibility by pioneer factors impact on the global gene expression patterns.*


 
**Reviewer #2:** “This paper describes a novel algorithm for the differential analyses of ATAC-seq profiles. The method is validated using various datasets from GEO relating to ATAC-seq profiles before and after the action of pioneer transcription factors. Validation of improvements with the SeATAC tool is mostly documented through detection of larger sets of differentially accessible regions. Further, these enhanced subsets are validated through Gene Ontology analyses for perceived biological relevance. This reviewer is a biologist, not a bioinformatician, and accordingly my comments originate from that viewpoint. The SeATAC tool appears to be an improvement compared to currently available methods, but the validation of the improvements provided by SeATAC is difficult to appraise from the bulk numbers provided. Indeed, it is not the number of sites but rather their biological relevance that needs to be conveyed; the GO analyses go only some way towards validation. “

Issue #1. The paper would be far more useful/convincing for biologists if it included a series of browser views for the sites that are detected with seATAC but not with other procedures in order to inform on the validity of the interpretations.

*Response: We thank the reviewer for these comments and agree with the reviewer that concrete examples will be helpful for the audience to compare and evaluate the performance of SeATAC and other tools. We provided examples of Etv2 binding sites with significantly altered chromatin accessibility during the Etv2 induced reprogramming in Supplementary Figure 11 [3]. Please see our response to Reviewer #1's Issue #2 for the details. It should be noted that NucleoATAC did not produce contrastive nucleosome signals at these Etv2 binding site between MEFs and D7 Flk+ cells or samples*

Issue #2. Further, as SeATAC appears to be unique in documenting decreased chromatin accessibility at promoters of regulated genes, this aspect would be particularly interesting to biologists and browser views would be very helpful to convince the readers. To be really helpful, these browser views should include other typical marks of promoters and enhancers such as H3K4me3, H3K4me1, H3K27ac, etc.

*Response: Please see our response to Reviewer #1's Issue #2 for a list of Etv2 binding sites with significantly altered chromatin accessibility during the Etv2 induced reprogramming, as well as the temporal H3K27ac changes over altered Etv2 binding sites. The Etv2 binding associated increasing accessibility was coupled with increased nearby H3K27ac signal, and a "dip" in the H3K27ac peak (Supplementary Figure 6b and Supplementary Figure 11a - 11e), as found in pioneer factor induced iPSC or neuronal reprogramming[10,11]. The Etv2 binding associated decreasing accessibility was coupled with decreased nearby H3K27ac signal (Supplementary Figure 6b and Supplementary Figure 11f - 11j).

We also included the examples of Etv2 binding induced decrease of chromatin accessibility on cis-regulatory element (CRE) near gene Brachury (T) and Mycn (Figure 6g and Supplementary Figure 6c).

To examine additional histone modification changes (e.g., H3K4me1 and H3K4me3) over transcription factor binding sites with altered chromatin accessibility, we compiled the ATAC-seq data from GM12878 and K562 cell lines [13,40], and used SeATAC to detect the nucleosome changes over NFKB1 binding sites, which are enriched in the GM12878 cell lines[41]. We identified 728 and 1,633 NFKB1 binding sites that had decreased chromatin accessibility in K562 in comparison with GM12878 (SeATAC p-value < 0.05 and log ratio > 0.2) in distal and promoter region, respectively (Supplementary Figure 5a). Similar to what we observed in the Etv2 induced reprogramming, there were "dips" of the signals at the NFKB1 binding sites of all three examined euchromatic marks (H3K4me1, H3K4me3 and H3K27ac) (Supplementary Figure 5b)

To further explore whether the local ATAC-seq signal captured by SeATAC, MACS2 or NucleoATAC can be predictive of the histone modification signals, we used the features produced by three tools to train a simple multilayer perceptron (MLP) to predict the H3K27ac, H3K4me1 and H3K4me3 signals. Similar with predicting differentially expressed genes from ATAC-seq signals over promoter region (see our response to Reviewer #1's Issue #1 and Supplementary Figure 4), we used the latent representations of the V-plots for SeATAC, and used principal components (PC) of the pileup signals (MACS2's pileup output) and the nucleosome signals (NucleoATAC's smooth signal output) to construct the features for MACS2 and NucleoATAC, respectively (Supplementary Figure 5c). We trained the MLP model on data from GM12878, and tested it on K562 (H3K27ac, H3K4me1 and H3K4me3), Etv2 induced reprogramming (H3K27ac only) and Etv2 induced EB differentiation (H3K27ac only). The mean squared error between known and predicted histone modification signals were used to quantitatively evaluate the prediction performance. SeATAC had the best performance of predicting 6 out of 7 histone modifications (Supplementary Figure 5d). In summary, along with other experiments included this manuscript, such as (1) detection of differential V-plot, (2) definition of nucleosome positions, (3) detection of nucleosome changes, (4) designation of TFBS with differential chromatin accessibility and (5) predicting of differential expressed genes, these results suggested that SeATAC could be used to more accurately detect various local genomic changes from the ATAC-seq data, including histone modifications.*

Issue #3. Finally, an attempt should be made to define the limitations of SeATAC, for example by comparisons with sites of biological relevance identified/discussed in the original papers that reported the datasets.

*Response: We agree with the reviewer and added one paragraph on the limitations of SeATAC in the Discussion section. We want to emphasize that SeATAC was designed as a tool to model the local ATAC-seq data (e.g., a 640 bp window) as a V-plot and to provide more accurate information regarding the local chromatin accessibility changes, such as nucleosome positions and nucleosome phasing [3,4]. However, SeATAC could not directly predict the global outcome (e.g. gene expression changes) based on the changes of local chromatin accessibility. Though SeATAC was able to determine a significant amount of pioneer factor induced decreasing of chromatin accessibility, the functional role of these events need to be confirmed by further experiments such as mutagenesis followed ATAC-seq or ATAC-PCR, especially for the distal enhancers such as the ZPA regulatory sequence (ZRS) in limb development [4].*

Issue #4. The terminology used to characterize the accessible domains identified by SeATAC is a bit simplistic, if not shocking, because it only offers a black and white, positive and negative, view of situations. For example, referring to NOR, nucleosome occupied regions, for promoters that become less accessible following the action of pioneers is inappropriate because these promoters show decrease in accessibility but do not become "fully occupied" as suggested by the wording. Since the ATAC measurements record DNA accessibility, it would be more appropriate to use acronyms that refers to DNA accessibility rather than to nucleosome occupied or free regions: the latter is a gross overinterpretation. Referring to increased or decreased accessibility would be more appropriate.

*Response: We agree with the reviewer that the term NOR (nucleosome occupied region) and NFR (nucleosome free region) may not accurately define the nucleosome status, especially when the relative nucleosome changes were evaluated, and the investigated regions may not be completely nucleosome-free or -occupied. In the revised manuscript, we replaced the term "NOR->NFR" by "increased accessibility", and "NFR->NOR" by "decreased accessibility" in the text, figures and figure legends. For task #2 and #3, however, the NOR and NFR was defined according to a full ATAC-seq data with stringent criteria (a genomic locus was considered as a NOR center if the NucleoATAC signal at this locus was greater than 0.5 and was also greater than any other positions in the flanking 200 bp region. A genomic locus was considered as a NFR center if the NucleoATAC signal at this locus was smaller than 0.01 and was also smaller than any other position in the flanking 200 bp region). The criteria ensured more accurate nucleosome status over specific loci. Thus, we believe that the terms NOR and NFR were still appropriate and necessary in task #2 and #3 (definition of nucleosome positions and detection of nucleosome changes).*

Issue #5. Minor comments
L.65 Protected or unprotected?
L.155 "Regarding".

*Response: We thank the reviewer for their comments, and we have corrected these errors in the revised manuscript.
 
References

1. Duren Z, Chen X, Xin J, Wang Y, Wong W. Time course regulatory analysis based on paired expression and chromatin accessibility data. Genome Res. 2020;30:gr.257063.119.
2. Duren Z, Chen X, Jiang R, Wang Y, Wong WH. Modeling gene regulation from paired expression and chromatin accessibility data. Proc Natl Acad Sci U S A. 2017;114:E4914–23.
3. Gong W, Das S, Sierra-Pagan JE, Skie E, Dsouza N, Larson TA, et al. ETV2 functions as a pioneer factor to regulate and reprogram the endothelial lineage. Nat Cell Biol. 2022;24:672–84.
4. Koyano-Nakagawa N, Gong W, Das S, Theisen JWM, Swanholm TB, Ly DV, et al. Etv2 regulates enhancer chromatin status to initiate Shh expression in the limb bud. Nat Commun. 2022;13:4221.
5. Kiani K, Sanford EM, Goyal Y, Raj A. Changes in chromatin accessibility are not concordant with transcriptional changes for single‐factor perturbations. Mol Syst Biol. 2022;18:e10979.
6. González AJ, Setty M, Leslie CS. Early enhancer establishment and regulatory locus complexity shape transcriptional programs in hematopoietic differentiation. Nat Genet. 2015;47:1249–59.
7. Anders S, Huber W. Differential expression analysis for sequence count data. Genome Biol. 2010;11:R106.
8. Wapinski OL, Lee QY, Chen AC, Li R, Corces MR, Ang CE, et al. Rapid Chromatin Switch in the Direct Reprogramming of Fibroblasts to Neurons. Cell reports. 2017;20:3236–47.
9. Wapinski OL, Vierbuchen T, Qu K, Lee QY, Chanda S, Fuentes DR, et al. Hierarchical mechanisms for direct reprogramming of fibroblasts to neurons. Cell. 2013;155:621–35.
10. Aydin B, Kakumanu A, Rossillo M, Moreno-Estellés M, Garipler G, Ringstad N, et al. Proneural factors Ascl1 and Neurog2 contribute to neuronal subtype identities by establishing distinct chromatin landscapes. Nature Neuroscience. 2019;22:897–908.
11. Chronis C, Fiziev P, Papp B, Butz S, Bonora G, Sabri S, et al. Cooperative Binding of Transcription Factors Orchestrates Reprogramming. Cell. 2017;
12. King HW, Klose RJ. The pioneer factor OCT4 requires BRG1 to functionally mature gene regulatory elements in mouse embryonic stem cells. eLife. 2017;
13. Buenrostro JD, Giresi PG, Zaba LC, Chang HY, Greenleaf WJ. Transposition of native chromatin for fast and sensitive epigenomic profiling of open chromatin, DNA-binding proteins and nucleosome position. Nat Methods. 2013;10:1213–8.
14. Zenere A, Rundquist O, Gustafsson M, Altafini C. Using high-throughput multi-omics data to investigate structural balance in elementary gene regulatory network motifs. Bioinformatics. 2021;38:173–8.
15. Li D, Liu J, Yang X, Zhou C, Guo J, Wu C, et al. Chromatin Accessibility Dynamics during iPSC Reprogramming. Cell Stem Cell. 2017;21:819-833.e6.
16. Knaupp AS, Buckberry S, Pflueger J, Lim SM, Ford E, Larcombe MR, et al. Transient and Permanent Reconfiguration of Chromatin and Transcription Factor Occupancy Drive Reprogramming. Cell Stem Cell. 2017;21:834-845.e6.
17. Yagi M, Ji F, Charlton J, Cristea S, Messemer K, Horwitz N, et al. Dissecting dual roles of MyoD during lineage conversion to mature myocytes and myogenic stem cells. Gene Dev. 2021;35:1209–28.
18. Wu L, Zhao G, Xu S, Kuang J, Ming J, Wu G, et al. The nuclear factor CECR2 promotes somatic cell reprogramming by reorganizing the chromatin structure. J Biol Chem. 2021;296:100022.
19. Benchetrit H, Jaber M, Zayat V, Sebban S, Pushett A, Makedonski K, et al. Direct Induction of the Three Pre-implantation Blastocyst Cell Types from Fibroblasts. Cell Stem Cell. 2019;24:983-994.e7.
20. Schwarz BA, Cetinbas M, Clement K, Walsh RM, Cheloufi S, Gu H, et al. Prospective Isolation of Poised iPSC Intermediates Reveals Principles of Cellular Reprogramming. Cell Stem Cell. 2018;23:289-305.e5.
21. Tang Y, Xiong S, Yu P, Liu F, Cheng L. Direct Conversion of Mouse Fibroblasts into Neural Stem Cells by Chemical Cocktail Requires Stepwise Activation of Growth Factors and Nup210. Cell Reports. 2018;24:1355-1362.e3.
22. Liu X, Ouyang JF, Rossello FJ, Tan JP, Davidson KC, Valdes DS, et al. Reprogramming roadmap reveals route to human induced trophoblast stem cells. Nature. 2020;586:101–7.
23. Liu Q, Jiang C, Xu J, Zhao M-T, Bortle KV, Cheng X, et al. Genome-Wide Temporal Profiling of Transcriptome and Open Chromatin of Early Cardiomyocyte Differentiation Derived From hiPSCs and hESCs. Circ Res. 2017;121:376–91.
24. Markov GJ, Mai T, Nair S, Shcherbina A, Wang YX, Burns DM, et al. AP-1 is a temporally regulated dual gatekeeper of reprogramming to pluripotency. Proc National Acad Sci. 2021;118:e2104841118.
25. Ramirez RN, El-Ali NC, Mager MA, Wyman D, Conesa A, Mortazavi A. Dynamic Gene Regulatory Networks of Human Myeloid Differentiation. Cell Syst. 2017;4:416-429.e3.
26. Maza I, Caspi I, Zviran A, Chomsky E, Rais Y, Viukov S, et al. Transient acquisition of pluripotency during somatic cell transdifferentiation with iPSC reprogramming factors. Nat Biotechnol. 2015;
27. Denny SK, Yang D, Chuang C-H, Brady JJ, Lim JS, Grüner BM, et al. Nfib Promotes Metastasis through a Widespread Increase in Chromatin Accessibility. Cell. 2016;166:328–42.
28. Zviran A, Mor N, Rais Y, Gingold H, Peles S, Chomsky E, et al. Deterministic Somatic Cell Reprogramming Involves Continuous Transcriptional Changes Governed by Myc and Epigenetic-Driven Modules. Cell Stem Cell. 2019;24:328-341.e9.
29. Corces MR, Buenrostro JD, Wu B, Greenside PG, Chan SM, Koenig JL, et al. Lineage-specific and single-cell chromatin accessibility charts human hematopoiesis and leukemia evolution. Nat Genet. 2016;48:1193–203.
30. Perrin HJ, Currin KW, Vadlamudi S, Pandey GK, Ng KK, Wabitsch M, et al. Chromatin accessibility and gene expression during adipocyte differentiation identify context-dependent effects at cardiometabolic GWAS loci. Plos Genet. 2021;17:e1009865.
31. Sanford EM, Emert BL, Coté A, Raj A. Gene regulation gravitates toward either addition or multiplication when combining the effects of two signals. Elife. 2020;9:e59388.
32. Qu K, Zaba LC, Giresi PG, Li R, Longmire M, Kim YH, et al. Individuality and Variation of Personal Regulomes in Primary Human T Cells. Cell Syst. 2015;1:51–61.
33. Landt SG, Marinov GK, Kundaje A, Kheradpour P, Pauli F, Batzoglou S, et al. ChIP-seq guidelines and practices of the ENCODE and modENCODE consortia. 2012;22:1813–31.
34. Ou J, Liu H, Yu J, Kelliher MA, Castilla LH, Lawson ND, et al. ATACseqQC: a Bioconductor package for post-alignment quality assessment of ATAC-seq data. Bmc Genomics. 2018;19:169.
35. Zaret KS, Carroll JS. Pioneer transcription factors: establishing competence for gene expression. Genes Dev. 2011;25:2227–41.
36. Soufi A, Garcia MF, Jaroszewicz A, Osman N, Pellegrini M, Zaret KS. Pioneer transcription factors target partial DNA motifs on nucleosomes to initiate reprogramming. Cell. 2015;161:555–68.
37. Zaret KS. Pioneer Transcription Factors Initiating Gene Network Changes. Annu Rev Genet. 2020;54:1–19.
38. Gualdi R, Bossard P, Zheng M, Hamada Y, Coleman JR, Zaret KS. Hepatic specification of the gut endoderm in vitro: cell signaling and transcriptional control. Gene Dev. 1996;10:1670–82.
39. Mayran A, Sochodolsky K, Khetchoumian K, Harris J, Gauthier Y, Bemmo A, et al. Pioneer and nonpioneer cooperation drives lineage specific chromatin opening. bioRxiv. 2019;472647.
40. Consortium EP, Bernstein BE, Birney E, Dunham I, Green ED, Gunter C, et al. An integrated encyclopedia of DNA elements in the human genome. Nature. 2012;489:57–74.
41. Buenrostro JD, Wu B, Litzenburger UM, Ruff D, Gonzales ML, Snyder MP, et al. Single-cell chromatin accessibility reveals principles of regulatory variation. Nature. 2015;*

**Second round of review**

**Reviewer 1**

The authors performed additional experiments and the new results fully addressed my concern. The major contribution is highlighted. I recommend its acceptance.
